# Evaluation of different digital elevation models for analyzing drainage morphometric parameters in a mountainous terrain: a case study of the Supin–Upper Tons Basin, Indian Himalayas

**DOI:** 10.1186/s40064-016-3207-0

**Published:** 2016-09-13

**Authors:** Sayantan Das, Priyank Pravin Patel, Somasis Sengupta

**Affiliations:** 1 Department of Geography, Bhairab Ganguly College, 2, Feeder Road, Belghoria, Kolkata, West Bengal 700056 India; 2 Department of Geography, Presidency University, 86/1, College Street, Kolkata, West Bengal 700073 India; 3 Department of Geography, Malda Women’s College, No. 3, Government Colony, Andigopal, Pirojpur, English Bazar, Malda District, West Bengal 732101 India

**Keywords:** SRTM, ASTER, Cartosat, Topographical maps, DEM, Morphometry, Accuracy

## Abstract

**Background:**

With myriad geospatial datasets now available for terrain information extraction and particularly streamline demarcation, there arises questions regarding the scale, accuracy and sensitivity of the initial dataset from which these aspects are derived, as they influence all other parameters computed subsequently. In this study, digital elevation models (DEM) derived from Advanced Spaceborne Thermal Emission and Reflection Radiometer (ASTER V2), Shuttle Radar Topography Mission (SRTM V4, C-Band, 3 arc-second), Cartosat -1 (CartoDEM 1.0) and topographical maps (R.F. 1:250,000 and 1:50,000), have been used to individually extract and analyze the relief, surface, size, shape and texture properties of a mountainous drainage basin.

**Results:**

Nestled inside a mountainous setting, the basin is a semi-elongated one with high relief ratio (>90), steep slopes (25°–30°) and high drainage density (>3.5 km/sq km), as computed from the different DEMs. The basin terrain and stream network is extracted from each DEM, whose morphometric attributes are compared with the surveyed stream networks present in the topographical maps, with resampling of finer DEM datasets to coarser resolutions, to reduce scale-implications during the delineation process. Ground truth verifications for altitudinal accuracy have also been done by a GPS survey.

**Conclusions:**

DEMs derived from the 1:50,000 topographical map and ASTER GDEM V2 data are found to be more accurate and consistent in terms of absolute accuracy, than the other generated or available DEM data products, on basis of the morphometric parameters extracted from each. They also exhibit a certain degree of proximity to the surveyed topographical map.

## Background

Topography is a key controlling factor in the operation of a variety of natural processes (Summerfield and Hulton 1994; Montgomery and Brandon 2002). Hence it needs to be quantitatively analyzed (Pike 2000; Lague et al. 2003), to ascertain the relative efficacy of its constituents and operative mechanisms (Brierley et al. 2006; Phillips 2007), and to gauge the response of geomorphic systems to different stimuli (Phillips 2006, 2009; Ahmed et al. 2010). Rivers are one of the most sensitive elements of the landscape (Brunsden 2001; Thomas 2001; Smedberg et al. 2009), and fluvial systems represent a long-term adjustment of streams (Whipple 2001; Tucker 2004), to the varying conditions of climate, lithology and tectonics (Burt 2001; Kirby and Whipple 2012; Whittaker 2012). Changes in the prevailing climatic conditions (Bogaart and van Balen 2000; Huisink 2000; Wobus et al. 2010), base levels (Blum and Tornqvist 2000; Stokes et al. 2002), and/or tectonic situations (Whipple 2004), may trigger short and long term responses by fluvial systems in the form of channel morphological adjustments (Rinaldi 2003), discharge and sediment regime changes (Whipple and Tucker 2002), and re-sculpting of the riparian landforms and landscape (Vandenberghe 2002; Nicholas and Quine 2007; Rittenour et al. 2007). These responses, particularly to structural disturbances and tectonic forcing, are usually manifested in the form of major anomalies in the morphometric attributes of rivers and their drainage network (van Heijst and Postma 2001; Church 2002; Lin and Oguchi 2006; Thomas et al. 2010, 2012; Bali et al. 2011; Bahrami 2013). Although recent researches have focused more on examining processes, materials and chronology (e.g. Lewin et al. 2005; Chiverrell et al. 2009; Hooke 2008; Trimble 2009; Solleiro-Rebolledo et al. 2011), the systematic evaluation of land surfaces and drainage characteristics remains a central theme in geomorphology (e.g. Cammeraat 2002; Minar and Evans 2008; Siart et al. 2009; Paik and Kumar 2010; Prasannakumar et al. 2013). Consequently, geomorphometry (i.e., the science of the measurement of landforms), occupies an important domain in the discipline (Rao 2002; Wobus et al. 2006; Bishop et al. 2012; Evans 2012).

This ‘geomorphometry’ may be classified into two types—‘general geomorphometry’, which analyses the overall land surface form, and ‘specific geomorphometry’, which examines the characteristics of individual landforms (Evans 2012). Widespread application of general geomorphometry, particularly in drainage basin analysis can be observed (e.g. Vorosmarty et al. 2000; Jordan et al. 2005; Lindsay 2005; Wood 2009; Hayakawa and Oguchi 2009; Cavalli et al. 2013). These morphometric properties of a drainage basin are the quantitative attributes of the landscape, derived from the terrain, the elevation surface and the drainage network (Goudie 2004), and include size, relief, surface, shape and texture attributes. Their calculation is the first step in geomorphometry and quantitative geomorphology. Evaluation of these parameters also provides a basis for ascertaining the structural and lithological controls inherent in the landscape, as well as understanding the tectonic history of the river basins under consideration (Ferraris et al. 2012; Jacques et al. 2014).

Digital elevation models (DEMs) have been frequently used for the above morphometric analysis of river basins through the extraction of topographic parameters and stream networks, and their use presents many advantages over traditional topographical maps. A DEM may be defined as a regular gridded matrix representation of the continuous variation of relief over space (Burrough 1986), and is a digital model of the land surface form. The primary requirement of any DEM is that it should have the desired accuracy and resolution and be bereft of data voids (Sefercik and Alkan 2009). Their steady and widespread application can be further attributed to their easy integration within a GIS environment (Moore et al. 1991; Weibel and Heller 1991). Before the year 2000, the base elevation models depicting a global coverage were available in a 1 km resolution like GTOPO-30 (Global Topography in 30 arc-sec) and GLOBE (The Global Land 1 km- Base Elevation Project) (Sefercik and Alkan 2009). However, in the last decade, more advanced global DEMs with better resolutions have become available, like the Shuttle Radar Topography Mission (SRTM) (version 4, C-Band DEM of 3 arc-second, 90 m resolution) and the Advanced Spaceborne Thermal Emission and Reflection Radiometer (ASTER) (version 2, 30 m resolution) DEMs which have mitigated the problem of spatial resolution to a great extent. For users within India, or those seeking to examine Indian landscapes, the available DEM dataset library received another member through the release of the CartoDEM data (version 1, only for Indian territories) at 30 m in 2011. Apart from these freely available ready-made DEM datasets, purchased stereo-images from a number of satellites (e.g. Cartosat 1, Landsat 7 ETM+, QuickBird, IKONOS, SPOT, ASTER sensors, among others) have also been used to create DEMs using various software applications for examining landscapes (Toutin et al. 2001; Toutin 2002, 2004; Poli et al. 2002; Hirano et al. 2003; d’Angelo et al. 2008; Deilami and Hashim 2011; Giribabu et al. 2013).

The biggest advantage of DEMs over traditional topographical maps is the seamless provision of data having a global coverage. Due to their wide applicability and ease of use, DEMs have been used in a variety of studies where terrain and drainage factors play prominent roles. Numerous studies on morphometric analysis from DEMs have been carried out across the world in recent years (e.g. Dietrich et al. 1993; Nag 1998; Snyder et al. 2000; Lindsay et al. 2004; Korup et al. 2005; Mesa 2006; Deng 2007; Ehsani and Quiel 2008; Lindsay and Evans 2008; Wilson et al. 2008; Wang et al. 2010; Ferraris et al. 2012; Caraballo-Arias et al. 2014; Jacques et al. 2014). In India, some prominent studies where DEMs have been employed for river basin analysis, estimation of soil loss, water resource evaluation and topographic characterization include Chopra et al. (2005), Kale and Shejwalkar (2007), Rudraiah et al. (2008), Sreedevi et al. (2009), Patel and Sarkar (2010), Malik et al. (2011), Pareta and Pareta (2011), Agarwal et al. (2012), Patel et al. (2012), Altaf et al. (2013), Magesh et al. (2012, 2013), Agarwal et al. (2013), Dar et al. (2013), Prabu and Baskaran (2013), Singh et al. (2013, 2014), Aher et al. (2014), Ambili and Narayana (2014), Magesh and Chandrasekar (2014), and Ghosh et al. (2015), among others. DEM usage in drainage routing and flood prediction too has gained popularity (e.g. Ozdemir and Bird 2009; Youssef et al. 2011; Sreedevi et al. 2013)

Normally it is accepted that higher resolution DEMs are more precise (Saran et al. 2009), and that this higher precision implies a greater degree of accuracy and a finer extraction of the land surface components, especially slope facets (Dragut and Blaschke 2006), and drainage lines (Anornu et al. 2012; Srivastava and Mondal 2012). Hence, the search for an optimal cell resolution and cell-size of DEMs has been a topic of research in the last few years (Hancock et al. 2006; Sharma et al. 2009; Sreedevi et al. 2009; Ahmed et al. 2010). In such geomorphometric analysis, the DEM resolution governs the scale of the features extracted (Hengl and Evans 2009), with the morphometric attributes extracted being also scale dependant (Dragut et al. 2009). A number of studies have delved into the accuracy assessment of individual DEM datasets, e.g. for SRTM data (Gorokhovich and Voustianiouk 2006; Weydahl et al. 2007), or for ASTER data (Eckert et al. 2005; San and Suzen 2005; Cook et al. 2012), and have looked into their effect on the extracted features like drainage (Fujita et al. 2008; Li and Wong 2010; Tarekegn et al. 2010) and terrain aspects (Zhou and Liu 2004; Vaze et al. 2010). Previous studies have also shown that the pre-release ASTER-GDEM had yielded better results than the SRTM-DEM in western Japan (Hayakawa et al. 2008); but its post-release version was found to be inferior to the SRTM-DEM in the mountainous regions of Turkey (Sefercik 2012). However, instances where two or more sets of DEMs were compared with respect to their morphometric parameters (e.g. Lindsay and Evans 2008; Taramelli et al. 2008; Hirt et al. 2010; Hosseinzadeh 2011; Suwandana et al. 2012; Mukherjee et al. 2013; Gopinath et al. 2014), are relatively few, especially in mountain landscapes of India. Furthermore, often in this accuracy assessment, the focus is more on comparison of absolute elevation parameters with lesser focus given to investigating how the various morphometric variables that are derived, vary from one dataset to the other, as well as how their prepared maps differ. Therefore, in this study, the morphometric properties of the Supin–Upper Tons watershed, located amidst the Garhwal Himalayas in India, are initially computed from different DEMs as well as topographical maps, and then subsequently mapped and compared in order to ascertain the most reliable source of digital elevation data for geomorphometry, generally overall and particularly for such regions.

This study compares elevation profiles, stream networks and morphometric parameters derived from freely available DEM products as well as from topographical maps of different scales for a chosen mountainous river basin. The comparison of these aspects, checked against field collected elevations at select Ground Control Points (GCPs) via a GPS survey, helped to ascertain which of the data products are more consistently able to represent the actual topographic features and are most useful in extensive drainage line demarcation, computation of stream statistics and enumeration of allied morphometric parameters. The study also highlights the extent of map scale or DEM resolution on terrain and drainage parameter extraction and how their respective maps differ as a result. To nullify this scale-effect, the different preliminary products were also re-sampled to a common resolution for better comparison and analysis, with the results tabulated.

## Study area

**Fig. 1:**
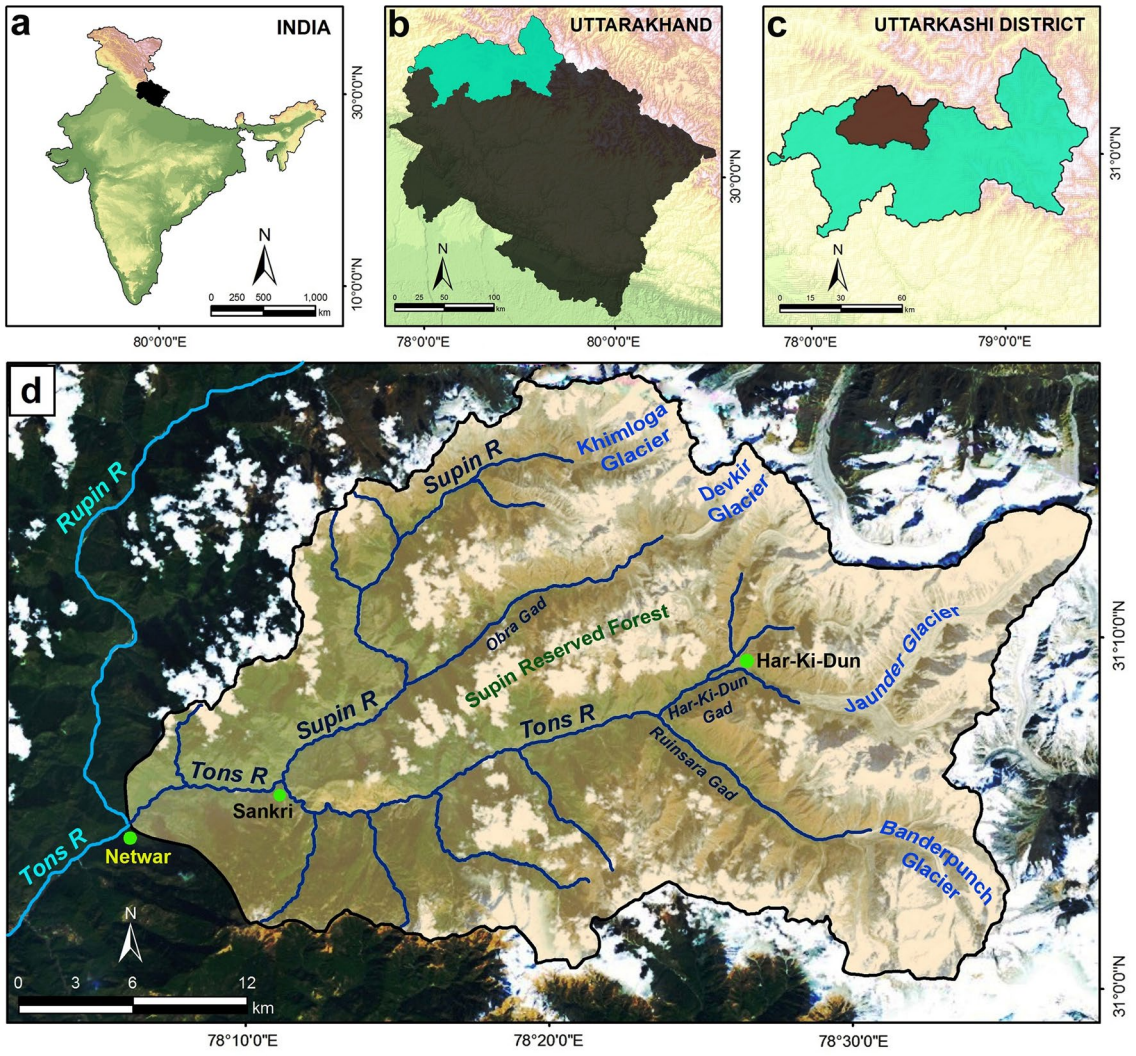
Location and geographical setting of the Supin–Upper Tons Basin along with important streams and glaciers. Inset panels show:** a** the location of Uttarakhand state in India,** b** the location of Uttarkashi district in Uttarakhand, and** c** the situation of the Supin-Upper Tons Basin in Uttarkashi. The basin boundary has been identified from the SoI topographical sheets and superimposed on a September 2012 Bing image (**d**). The important places like Netwar (Rupin-Tons confluence, termination point of the basin), Sankri (Supin–Tons confluence) and Hari-Ki-Dun have also been shown

The Supin–Upper Tons Basin comprises part of the Tons River (the largest tributary of the Yamuna River) Basin in the Purola Tehsil of the Garhwal Region of Uttarakhand state, India, located between 78°06′E–78°38′E and 31°00′N–31°17′N. The River Supin is one of the principal tributaries of the Tons River, which itself is a tributary of the Yamuna River (Pankaj et al. 2012). The other major tributary, the Obra Gad, merges with the Supin River near the village of Fitari. The Tons River is initially formed by the joining of its tributaries, the Har-ki-dun Gad and the Ruinsara Gad, before their combined flow meets the Supin River (Fig. 1). The basin covers an area of about 977 sq km approximately, having a perimeter of about 180 km. The Supin River itself originates from the snout of the Khimloga Glacier while the main stream, the Tons River, emerges from the Banderpunch Glacier and these rivers converge near Sankri village, about 30 km downstream from their respective sources. The basin mouth is near Netwar village where the combined flow of the Supin–Tons merges with the Rupin River.

The physiography of the watershed is dominated by high mountain ranges and steep spurs alternating with deep declivities, i.e. a landscape of sharp divides and entrenched river courses. The altitude ranges from 1200 m to 6387 m (Krishan et al. 2009). A substantial part of the basin is over 4000 m elevation above mean sea level. The highest point in the basin is the Banderpunch Peak (6315 m). The areas above 3000 m are more or less glaciated. Forests, agricultural tracts, snow covered hillslopes, glaciers and grasslands are the major land cover and land use types.

The rainfall received over the basin area varies from 1000 to 1500 mm annually, with occasional heavy cloud bursts and the area is subjected to regular snowfall, with significant amounts occurring between October to May (Krishan et al. 2009). Approximately 49 % of the entire basin area, especially its upper reaches, is under perennial snow cover (as ascertained from the IRS-P6 LISS-III image of the study area of October 2008, obtained from the Bhuvan Portal, after digitisation of the visible snow cover extent, and also subsequently verified from the USAMS and SoI topographical sheets). Past glacial retreat may be inferred from the present ‘U’ shaped valleys with moraines and aggradational slopes present, downstream from the present glacial snouts.

Geologically, the rocks exposed within the basin range from the Proterozoic to the Cambrian in sequence and age. The younger rocks occupy the northern and eastern parts while the older Proterozoics are divided into a number of tectonic groups in the west and south (GSI 2004). The area is cut across by a number of thrusts, namely the Purola Thrust, Main Central Thrust and Jutogh Thrust, which indicate the dynamic pressures the rocks have been subjected to and account for the large varieties of metamorphics seen here. Near the basin mouth, the Jaunsar Group comprises of rocks of Neo Proterozioc age with constituents like grey and green phyllites, quartzites and schists. The Purola Crystalline Group thrusts over the Jaunsar Group (via the Purola Thrust), in the lower basin portion, and contains amphibolite, pebbly conglomerate gneiss, biotite schists and quartz. The Central Crystalline Group, occupying the middle part of the basin, can be divided into lower grade and higher grade categories. The lower grades (called the Gangar Formation), comprise of inter-calated sequences of schists, mica, quartzites, biotites, quartz and gneiss. The higher grades (called the Har-ki-dun Formation) thrust over these lower grades (via the Main Central Thrust), and comprise of schist, gneiss, migmatites and basic intrusives. Emplacement of biotite granite (Rakcham Granite of Palaeozioc age) has also occurred in the central part of the basin. The eastern and northern portions comprise of the relatively younger Haimanta Division of the Early Cambrian rocks, which is further divisible into the Batal Formation and the Kunjanla Formation. The main rock types in these formations are grey phyllite, quartzite, carbonaceous shale and green shale. Intrusives of metamorphosed granite of the Paleozoic Era are also present in this area. The general dip of the rocks is NW–SE (Pankaj et al. 2012), and these have been subjected to intense deformation in the form of folding, thrusting and faulting, disrupting the original stratigraphic position of the various lithounits (GSI 2004).

Since a mountainous terrain has been chosen as the study area, ambiguities related to drainage extraction are expected to be absent. In a flat terrain, the drainage networks derived usually show wide deviations from reality (Rahman et al. 2010).

## Datasets and methods

Traditionally, morphometric attributes of drainage basins have been estimated manually by stream network and contour extraction from topographical maps. However, the degree of drainage elaboration on a topographical map is certainly scale-dependent, thereby restricting their use in the micro-geomorphic analysis of stream networks. The most-used maps in the Indian subcontinent are 15′ × 15′ sheets (R.F.1: 50,000), that have the following limitations:

- Contour-crenulations suggest valleys as they run through the contour V’s pointing headward; however, stream channels are not always drawn through them. Similarly, the headward limit of streams often truncate abruptly, although contour-crenulations seem to suggest further head-ward extension of these.
- Problems arise in mapping the channel network, especially when some disappear as they reach the foothill zones or due to cultivation across and along the channel beds.
- Errors may occur in delineating the stream course downstream of dams, if the reservoir stretches across two or more map sheets and any one of them is too dated to record the existence of the dam, that was constructed after the map was published.
- Some contours terminate at a map’s edge and are not carried over into the neighboring one, especially when contour intervals vary between adjacent maps.

These problems may be minimized by using a dataset of continuously distributed elevation data across an area. Moreover, the possible errors that may occur due to inaccurate channel network demarcation, masking effects of vegetation or cultivation and cartographic compulsions in map preparation, may be minimized by employing such continuous elevation data. Modern day DEMs have come as an answer to these issues.

This analysis of DEM derived information in the present study is thus topical and of importance as it influences analysis of landscape configuration. This paper provides a comparative study of different available or derived DEMs (from SRTM, ASTER, Cartosat-1 tiles and SoI, USAMS topographical maps), through extraction of stream networks and terrain aspects, enumeration of different morphometric indices, and their eventual comparison.

In this paper, DEMs derived from ASTER, SRTM, Cartosat-1 and topographical maps (R.F. 1:250,000 and 1:50,000) have been used to analyze separately the relief, surface, size, shape and texture properties of the aforementioned study area. The salient characteristics of these datasets are as follows:

- A map from the United States Army Map Service (USAMS) topographical map series (R.F. 1:250,000) has been used for the current study. This particular map was compiled in 1954 by the USAMS from the Half-Inch Series (R.F. 1:126,720) maps [53 I/SE (1946), 53 M/SW and 53 M/SE (1936)] and the Quarter-Inch Series (R.F. 1:253,440) maps [H 44A (1920), H 44B (1905)] produced by Survey of India (SoI).
- The SoI topographical maps (Metric Edition, map sheet numbers 53I/4, 53I/7, 53I/8, 53I/12) at 1:50,000 scale have been used for preparation of the base map. These maps were prepared on the basis of the surveys carried out in 1962-63.
- The Shuttle Radar Topography Mission (SRTM) that took place in February, 2000 was the first endeavor to compensate for the lack of a worldwide high-resolution DEM. The spatial resolution of this DEM is 3 arc-second which corresponds to about 90 m distance on the earth’s surface (USGS 2004; Sefercik and Alkan 2009). Heights are referenced to the WGS84 geoid in metres and data voids are assigned a value of −32,768. This gridded elevation data is available for all land between 60°N and 56°S latitudes.
- The Advanced Spaceborne Thermal Emission and Reflection Radiometer Global Digital Elevation Model (ASTER GDEM) dataset was made available for scientific and academic usage on and from June 29, 2009 (USGS and Japan ASTER Program 2003; Sefercik 2012). It covers land surfaces between 83°N and 83°S and is composed of 22,600 1° × 1° extent tiles. It is available in GeoTIFF format (*.tiff files), with geographic latitude/longitude coordinates and a 1 arc-second (30 m) grid of elevation postings. Referenced to the WGS84 geoid, ASTER GDEM is the largest DEM that covers the entire planet surpassing even the SRTM data set.
- The Cartosat-1 Digital Elevation Model (CartoDEM version 1) is an Indian DEM developed by the Indian Space Research Organization (ISRO). It is derived from the Cartosat-1 stereo payload launched in May 2005. The primary output unit is a tile of 7.5ʹ × 7.5ʹ extents with DEM spacing of 1/3 arc-sec, and co-registered ortho-image of resolution 1/12 arc-sec (ISRO and NRSC 2011). The CartoDEM is a surface model of elevation and covers land surfaces within India.

The general information about the different data sources used have been presented in Table 1.

**Table 1:** Details of the maps, satellite images and digital elevation models used in this study

Sl. No.	Producing authority	Details of the maps, satellite images, DEM Datasets	Year of survey /date of pass	Scale /spatial resolution
1	United States Army Map Service (USAMS)	Map no. NH-44 1, Series U502 Edition 1, compiled in 1954 from Half-inch Series and Quarter-inch Series Map produced by Survey of India (actual surveyed network)	1902–1946	1:250,000
2	Survey of India (SoI)	Map nos. 53I/4, 53I/7, 53I/8, 53I/12 (actual surveyed network)	1962–1963	1: 50,000
3	National Remote Sensing Centre (NRSC), India	IRS LISS 3, Path-096 Row-049; IRS LISS 3, Path-097 Row-049	23 October 2008	23.5 m
4		CartoDEM (produced from Cartosat-1 by ISRO) : 78E31 N–79E32 N	26 February 2008	30 m
5	National Aeronautics and Space Administration (NASA), USA	ASTER GDEM : N31E078	17 October 2011	30 m
6		SRTM DEM : N31E078	11 February 2000	90 m
7	Bing image Data source: www.bing.com/maps Imaging date information from: http://mvexel.dev.openstreetmap.org/bing/	Worldview - 1	September 2012	1 m
8	Generated DEMs	From Map no. NH-44 1, Series U502 Edition 1	–	From 1:250,000 maps
9		From Map nos. 53I/4, 53I/7, 53I/8, 53I/12	–	From 1: 50,000 maps
10		Resampled from 30 m ASTER GDEM : N31E078	–	90 m
11		Resampled from 30 m CartoDEM: 78E31 N–79E32 N	–	90 m

Processing and ensuing analysis of the above datasets has been performed sequentially using the following methods.

### DEM processing and extraction of drainage networks

**Fig. 2:**
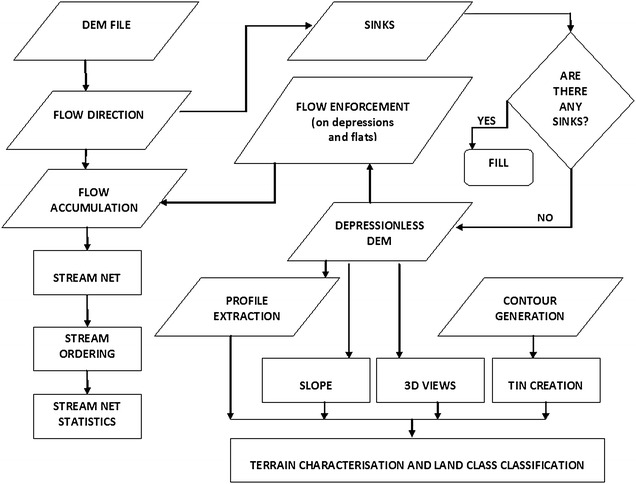
Flowchart showing the general process of extraction of a drainage basin using a DEM and subsequent processing leading to terrain characterization and land classification

A flowchart schematically shows the methodology followed  for the extraction of drainage networks and surface attributes from DEMs in a GIS environment (Fig. 2). The SRTM DEM of the study area is first preprocessed through the operations of *filling the data gaps*, *pit removal*–*depression filling,* and *finding outlet cells* in an iterative manner (O’Callaghan and Mark 1984; Jenson and Domingue 1988). *Pit removal* and *depression filling* is a method of filtering the digital elevation data. This is done to overcome any data voids that may be present in the DEM tile and to also ensure proper channel network connectivity. Sometimes, there are some pixels in the continuous array of digital data where the value of the pixel is abnormally low or high in comparison to other neighbouring cells. These are known as data sinks or spikes respectively and these are inherent in any DEM. These need to be removed before carrying out any sort of analysis in the data (Wood 1996). Along with the SRTM data, ASTER and Cartosat-1 DEMs were also preprocessed and all the possible data sinks and spikes were removed.

**Fig. 3:**
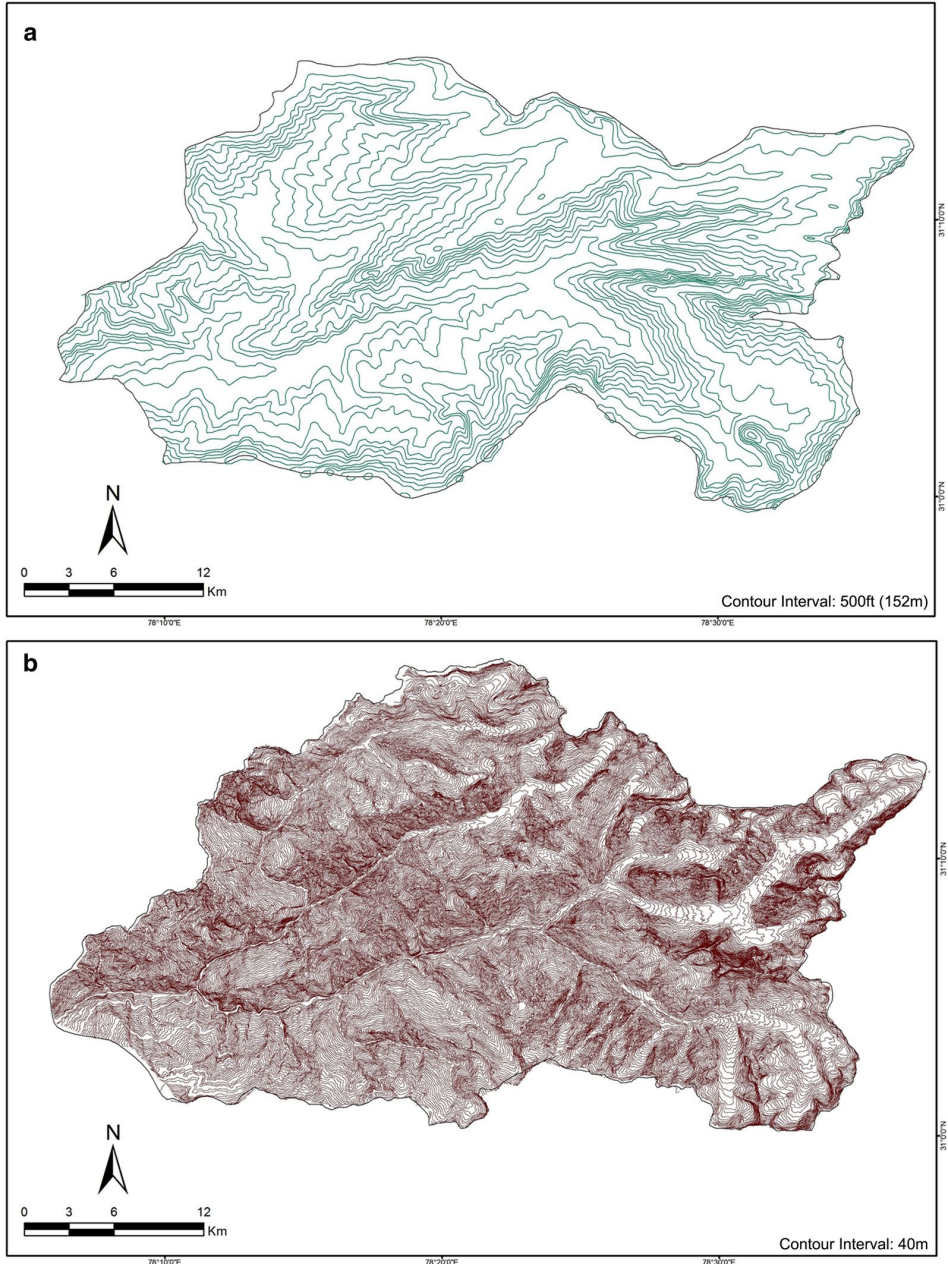
Contour lines have been digitized from USAMS topographical Sheet **a** and SoI topographical sheets **b** to derive the surface models in the form of DEMs. These DEMs have been used to obtain the drainage network within the these basins and the results have further been compared with the other DEMs like SRTM, ASTER and Cartosat-1

**Fig. 4:**
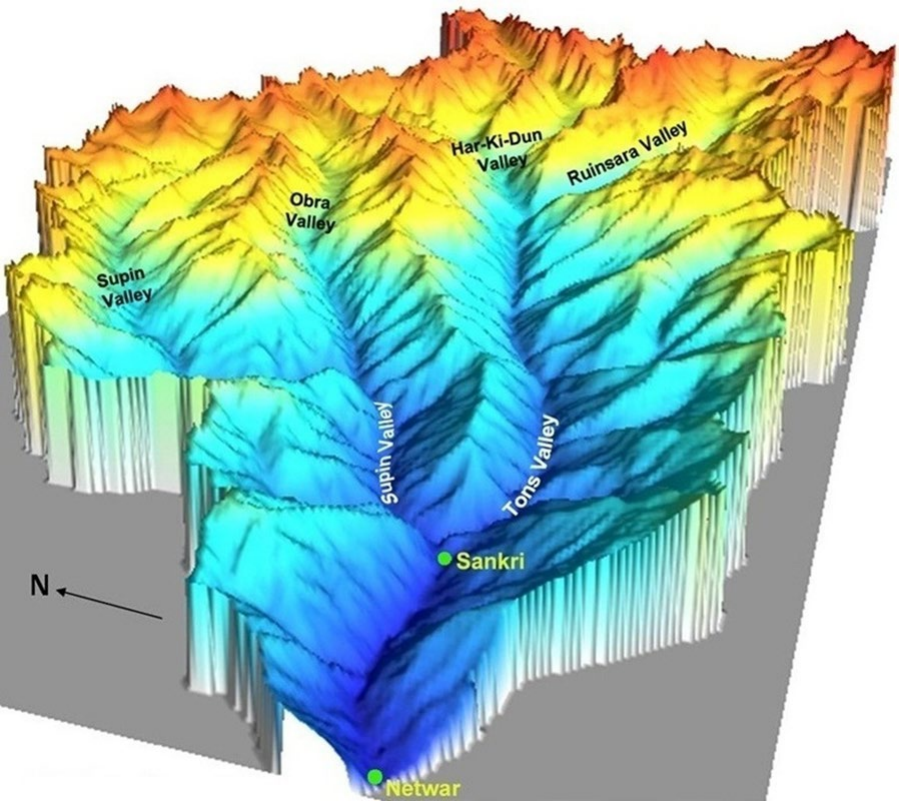
A 3-dimensional perspective of the surface model generated from SoI contours. The exaggeration factor is 1×

The derivation of DEMs from USAMS and SoI topographical maps involved a rather time-consuming and labour-intensive technique. These maps were obtained either as a scanned raster object (in case of the USAMS Map of scale 1:250,000) or as hard-copy maps that were then scanned at 300 dots per inch (in case of the SoI maps of scale 1:50,000). These were then georeferenced using the location information (latitude and longitude) demarcated in them. The contour lines were then digitised on-screen manually in the ArcGIS environment to prepare contour maps (Fig. 3). All such vector contour datasets were converted to WGS84 datum and then processed to derive the respective surface models (Fig. 4) through triangulated interpolation and subsequent smoothening of the derived surfaces. From these surface models, the stream networks were later extracted for the respective topographical maps, as described below.

A stream network develops as an interface between the concentrative processes acting in and towards the channels, as compared to the diffusive processes acting divergently, across the surrounding hill slopes. The simplest method for specifying flow directions is to assign flow from each pixel to one of its eight neighbors, either adjacent or diagonally, in the direction of the steepest downward slope. This method, designated D8 (choosing any 1 out of 8 flow directions on the basis of the line of steepest descent), was introduced by O’Callaghan and Mark (1984) and has been widely used as it generates channel networks with no divergence, allowing water to be routed unambiguously (Band 1986). In the context of a grid, the *upslope area* (A) contributing to each pixel is estimated as the product of the number of pixels draining through each pixel and the pixel area. The *specific catchment area* (SCA) is then estimated as *A/L*, taking *L* as the pixel width (Lindsay 2009). A pointer data layer is then created that stores the flow direction of each cell in a raster grid and the topology of the flow network is thus generated (Patel and Sarkar 2009). Within the GIS environment, algorithms for flow accumulation, flow routing and flow direction analysis were run which helped to extract the drainage network. This drainage network was then ordered using the Strahler (1954) scheme of stream ordering, wherein each of the finger-tip tributaries were designated as Order 1. Where two streams of the same order meet, the resulting stream order of the subsequent unified stream increases by one. This scheme was followed to categorise the streams derived from each of the DEMs.

It is pertinent to mention here that a portion of the studied basin remains under snow cover perennially. Therefore, stream network generation from the entire DEM creates streams over areas covered by glaciers, mostly by taking potential flow-lines along either edge of the flat ice-filled valley floor or through the base of the cliffs on either valley side, where they abut onto the edge of the glacier. This causes flow-lines to be shown where streams are placed parallel to each other till they join at the glacial snout to become a single stream segment. Such a derived network is erroneous as it greatly increases the network extent spuriously and also causes inaccuracies in stream ordering and subsequent evaluation of morphometric parameters. Therefore, an ice cover mask has been used initially, to allow stream extraction only in the ice free region and a more realistic drainage network is obtained for each of the analyzed datasets. This mask was demarcated via on-screen digitisation of the snow cover, as visually interpreted from an IRS P6 LISS-III scene of October 2008, imaged over the study area, and reconfirmed from the snow cover and glacial portions demarcated in the SoI topographical sheets. This digitised polygon layer was overlain on each of the Basin DEM surfaces, to mask out the portion covered by ice, in order that spurious and parallel channel networks were not derived over and along each side of the glacial snouts, which would then skew stream ordering and network length enumerations. Furthermore, to show the amount of error that non-usage of the ice-mask creates in extraction of the drainage network and its allied morphometric parameters, a comparison has also been laid out between the extracted flowlines from the overall area (i.e., obtained without using an ice-mask and thus erroneous in overestimating drainage line number and lengths) and from just the ice-free area (more realistic and accurate).

**Fig. 5:**
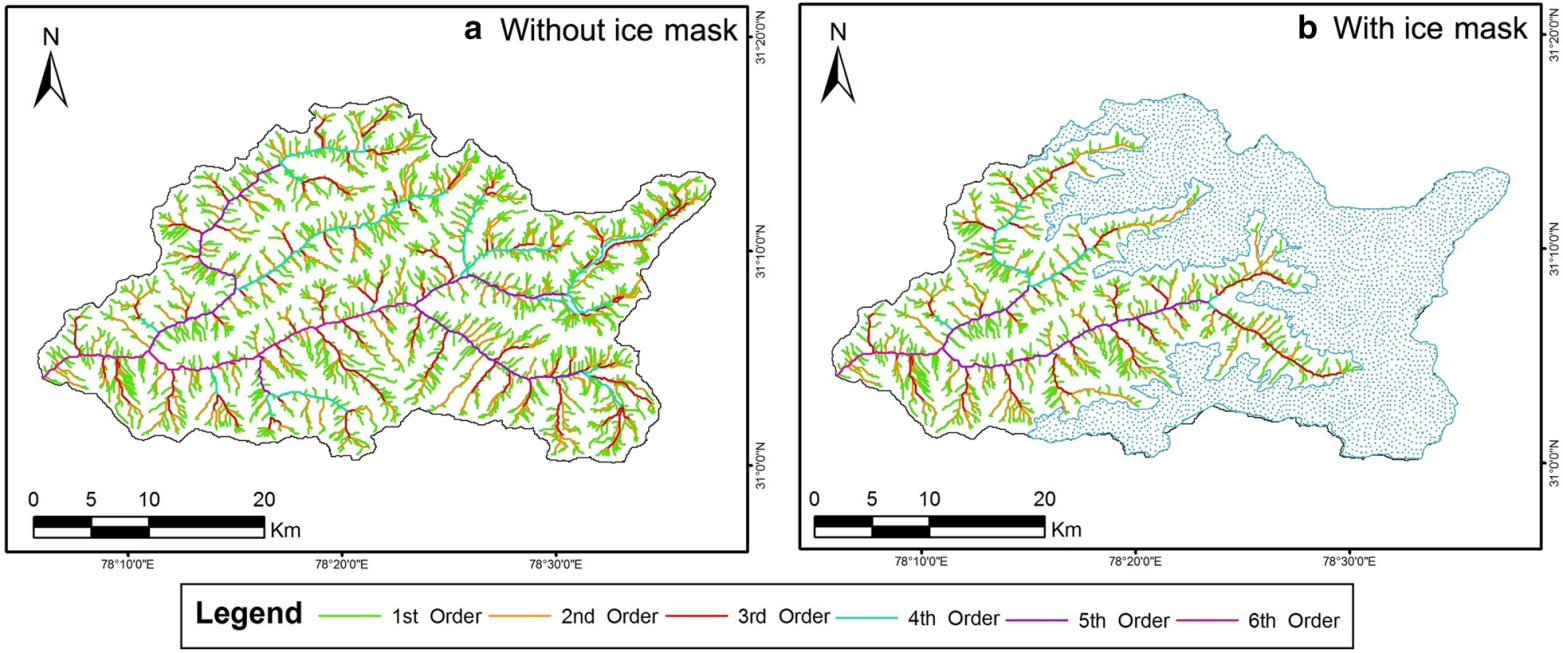
Stream networks extracted from SRTM DEM before (**a**) and after (**b**) employing the ice cover mask technique. Both the depictions reveal the study area as a 6th order drainage basin

**Fig. 6:**
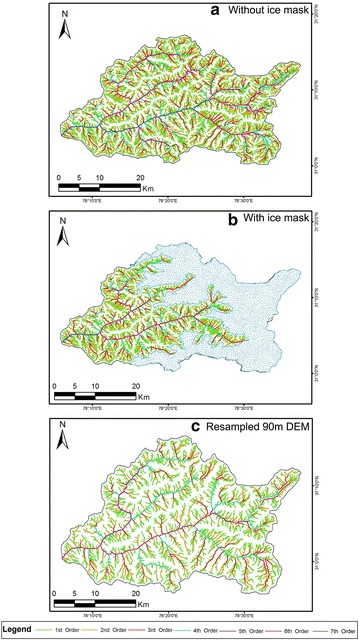
Stream networks extracted from ASTER GDEM before (**a**) and after (**b**) employing the ice cover mask technique alongside the resampled 90 m DEM (**c**). For the first two instances (**a**, **b**), the study area is a 7th order basin while the last illustration (**c**) reveals it to be a 6th order basin

**Fig. 7:**
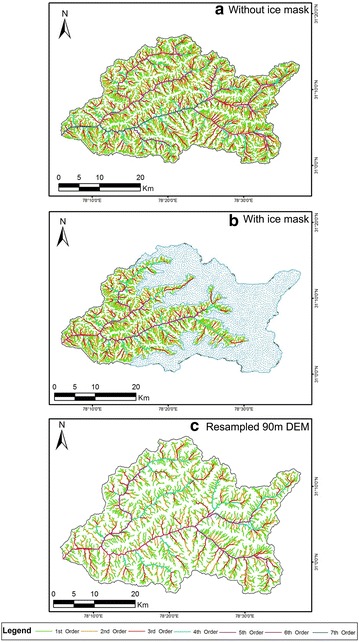
Stream networks extracted from Cartosat-1 DEM before (**a**) and after (**b**) employing the ice cover mask technique along with the resampled 90 m DEM (**c**). The first diagram (**a**) identifies the study area as a 7th order basin, whereas the other illustrations (**b**, **c**) recognize it to be a 6th order basin

Figures 5, 6 and Fig. 7 represent the drainage network of the Supin–Upper Tons Watershed, as extracted from SRTM, ASTER and Cartosat-1 DEM respectively—with and without the ice-cover mask and also for the resampled DEMs. Since the SRTM-DEM data has a resolution of 90 m and the ASTER and Cartosat-1 datasets are of 30 m resolution, the ASTER and Cartosat-1 datasets were resampled down to 90 m, to remove any bias in network derivation and subsequent computations in channel parameters that may occur due to this variation in resolution. Stream networks have been again subsequently re-derived for comparison from these resampled DEMs (Figs. 6c, 7c respectively).

**Fig. 8:**
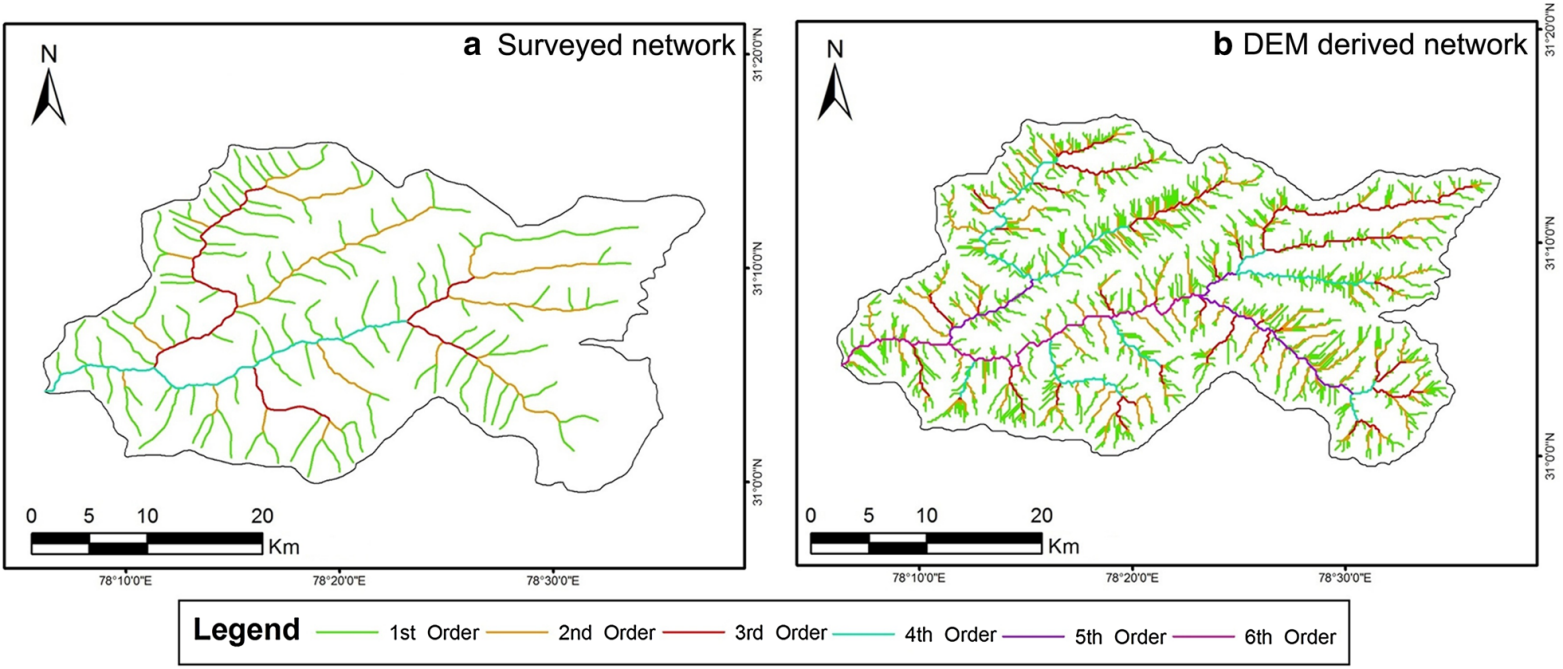
Surveyed stream networks from the USAMS topographical sheet (**a**) and DEM derived (obtained from surface modeling of digitized contour lines and spot heights of the USAMS topographical sheet) drainage networks (**b**). The first diagram (**a**) identifies the study area as a 4th order basin, whereas the other diagram (**b**) depicts it to be a 6th order basin. The masking technique has not been employed due to the non-appearance of ice cover within the map itself

**Fig. 9:**
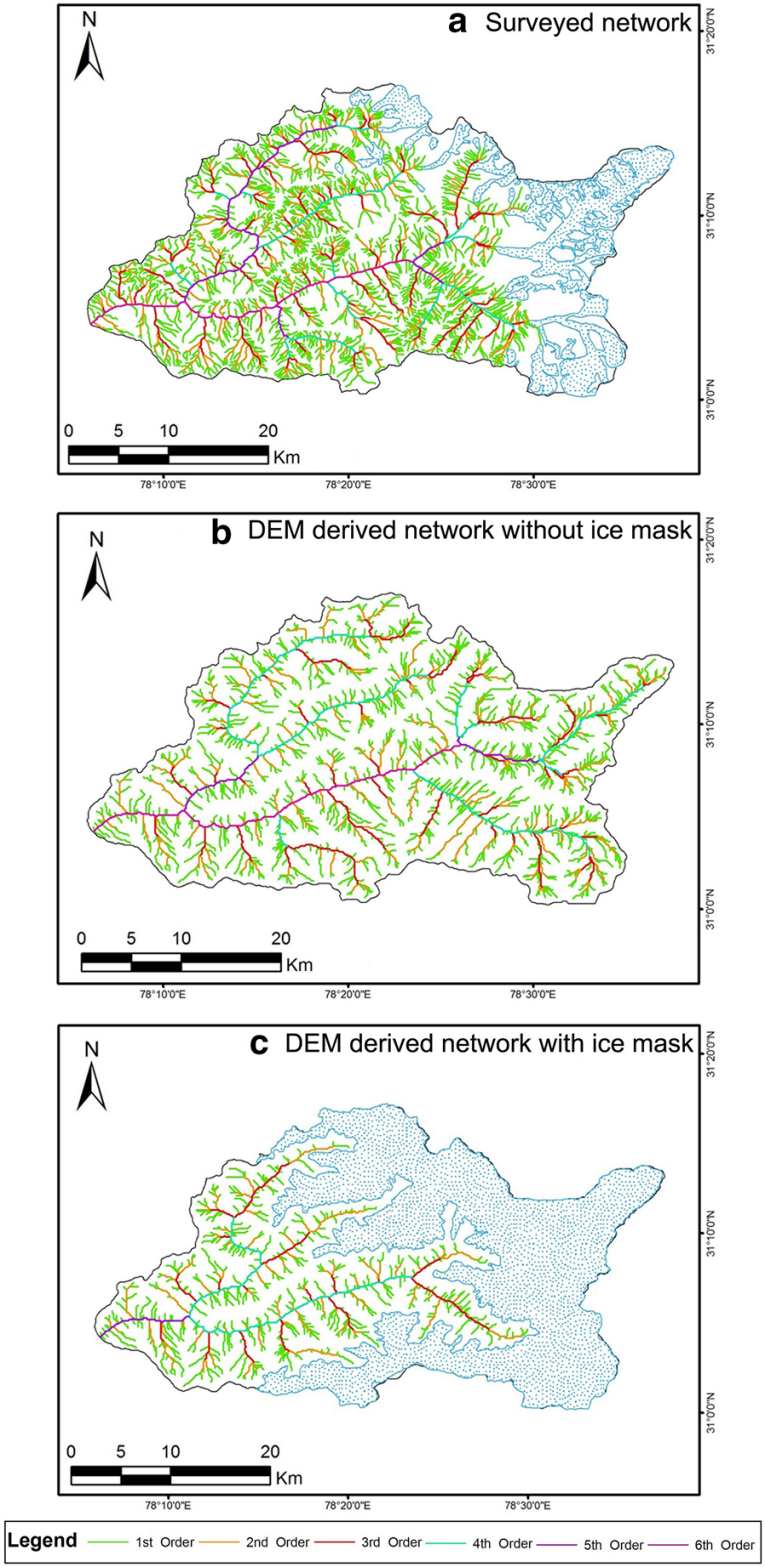
Surveyed stream networks from the SoI topographical sheets (**a**) along with DEM derived (obtained from surface modeling of digitized contour lines and spot heights of the SoI topographical sheet) drainage networks, before (**b**) and after (**c**) employing the ice cover mask technique. The study area is a 6th order basin as revealed from the first two diagrams (**a**, **b**) while the last diagram depicts it as a 5th order basin. The ice covered area is quite prominent in these topographical maps and the surveyed networks are devoid of any kind of inflated representations that normally occurs during the extraction of drainage networks from DEMs, if the ice cover is present in a drainage basin

The next exercise was to extract the stream network from the digitized contours of the USAMS and SoI topographical maps. The contours and all spot elevations were digitized, their respective elevation values input and these were then converted into a DEM via triangulated interpolation, with the resultant data being processed to extract the drainage networks as was done from the satellite-derived DEMs before (Figs. 8, 9). All the above datasets were then brought into a common reference framework (projection and datum-wise) for comparison. In total thus, there are nine separate datasets (3 downloaded DEMs, 2 resampled DEMs from finer data, 2 DEMs prepared via contour digitisation from topographical maps and 2 surveyed topographical maps), from which the various terrain and drainage attributes are subsequently derived and mapped for comparison. The notations used to refer to these datasets in this paper are as follows: A30 (ASTER 30 m DEM), A90 (ASTER resampled 90 m DEM), C30 (CartoDEM 30 m DEM), C90 (CartoDEM resampled 90 m DEM), S90 (SRTM 90 m DEM), T50A (actual digitised contour and stream network database from 1:50,000 scale topographical maps), T50D (DEM generated from digitised contours of 1:50,000 scale topographical maps, resampled to 90 m), T250A (actual digitised contour and stream network database from 1:250,000 scale topographical maps), and T250D (DEM generated from digitised contours of 1:250,000 scale topographical maps, resampled to 90 m). The basin outlines for each dataset (as presented within the foregoing figures), were also extracted in a GIS environment after demarcation and ordering of the channel network using the pour point function, wherein all the cells which drain into a particular outlet are grouped together and their combined perimeter forms the basin boundary. The areas of the basin polygons derived from each dataset thus, were then computed.

### Morphometric parameters extracted from the DEM and topographical map derived drainage networks and elevation surfaces

A number of morphometric parameters were enumerated for the respective stream networks and basins, derived from the different DEM and topographical map datasets outlined before. These were then compared in order to ascertain the most reliable source of digital elevation data for geomorphometric analysis of this basin. A brief description of the enumerated parameters is as follows:

- Catchment properties—These pertain to the geometric attributes of the respective stream networks and the basin polygons. The variables enumerated under this are:

i. Basin area (A): The areal extent of each basin
ii. Basin perimeter (P): The length of the circumference of each basin
iii. Basin length (L): The longest straight-line distance from the basin mouth to the basin boundary in its uppermost reach
iv. Main channel length (MCL): The length of the highest Strahler-order stream segment in the basin
v. Total channel length (TCL): Sum of lengths of all the stream segments within the basin
vi. Total number of stream segments (TSS): Sum of all the stream segments of all the Strahler orders in the basin
vii. Strahler order (SO): The highest Strahler-order stream in the basin, which gives the basin its order

- Relief properties—These pertain to the distribution of elevation points within the basin area. The variables enumerated under this are:

i. Maximum relief (MaxR)—The highest elevation point within the basin area
ii. Minimum relief (MinR)—The lowest elevation point within the basin area
iii. Mean relief (MR)—Average value of all the elevation points within the basin area
iv. Relative relief (RR)—Difference in elevation between the highest and lowest elevation points (RR = MaxR − MinR) (Smith 1935). High RR is indicative of youthful basins.
v. Relief ratio (RelR)—The basin relative relief normalised by the basin length (RelR = RR/L) (Morisawa 1965)
vi. Hypsometric integral (HI)—Derived from the relative area–altitude distribution within the basin, it indicates the proportionate volume of the basin still to be eroded (Strahler 1952). High HI is indicative of youthful basins.

- Shape properties—These pertain to the planar configuration of the basin outline. The variables enumerated under this are:

i. Circularity ratio (CR)—This compares the area of the basin to the area of a circle of the same circumference (CR = 4πA/P^2^) (Miller 1953). A perfectly circular basin returns a value of 1 while the value for an elongated basin tends towards 0.
ii. Elongation ratio (ER)—This compares the longest dimension of the basin (from the mouth) to the diameter of a circle of the same area as the basin (Schumm 1956)

- Texture properties—These pertain to a combination of the relief properties and the stream network attributes and show how the basin landscape is being successively eroded by the drainage lines. The variables enumerated under this are:

i. Stream frequency (SF)—Number of streams per unit area (SF = TSS/A) (Horton 1945). Higher SF values are indicative of less resistant rocks, which may aid greater erosion in the basin.
ii. Drainage density (DD)—Total length of streams per unit area (DD = TCL/A) (Horton 1945). Higher DD values indicate greater dissection of the basin surface and more potentiality for erosion.
iii. Constant of channel maintenance (CCM)—Reciprocal of DD, it indicates the amount of catchment area required for unit length of a stream to sustain its flow. This value is larger for arid regions. (CCM = 1/DD) (Horton 1945)
iv. Bifurcation ratio (R_b_)—Shows the average ratio at which streams of an order join those of the next higher order (Strahler 1954). Higher R_b_ values are indicative of greater structural control in the network.

- Grid-wise morphometric parameter extraction and mapping—Apart from examining the terrain and stream attributes of the overall drainage basin through the above basin-level parameters, it is further analysed how morphometric parameters differ across the different datasets when enumerated grid-wise over the basin surface. For this, the various DEM or digitised contour files for each dataset along with their corresponding basin perimeter file were overlain by a mesh of 1 km × 1 km dimension grids (1154 grids in all), and select morphometric parameters pertaining to elevation, relief and drainage attributes were evaluated for each grid, as follows:

i. Maximum, minimum and average elevation of each grid (derived on basis of DEM pixels or contour lines falling within each grid area)
ii. Relative relief for each grid (extracted as described before on basis of the highest and lowest elevation value for each grid).
iii. Slope (SLP): Extracted either directly from DEMs using relevant algorithm available in the software interface or through using Wentworth’s (1930) formula for topographical maps (SLP = No. of contour crossings per unit length × contour interval/636.6)
iv. Drainage density (DD)—The total length of streams within each grid, clipped accordingly, (Horton 1945). Higher DD values indicate greater dissection of the basin surface and more potentiality for erosion.

The above parameters, extracted for each grid, from the 1:50,000 scale topographical maps were taken as fixed or as reference values, against which the same parameters derived from the other DEM datasets were then compared. Thereafter, the respective differences for each parameter, on basis of their values derived from each of the other eight datasets for these grids, were computed, by subtracting its value from the corresponding topographical map value. A number of isopleth maps, through interpolation of the gridded morphometric parameters, were then prepared to visually represent this difference, keeping similar ranges to aid comparison, in order to find that particular dataset which most closely matched (in terms of actual values and the isoline trends), the surveyed large-scale topographical map derived values by showing the least deviation from the same. This dataset could then be claimed to best represent this terrain for these parameters and be used subsequently in further geomorphometric computations. The percentage of difference for each parameter was also computed grid-wise by dividing the above difference by the corresponding topographical map derived value for that grid, and then multiplying by 100. From these, the mean difference, standard deviation and coefficient of variation of this percentage difference between the different values, were finally computed.

## Results and discussion

### Variations in basin morphometric attributes extracted from the different datasets

The morphometric properties of the Supin–Upper Tons Watershed extracted from the different DEMs have been calculated and subsequently presented in Table 2.

**Table 2:** The morphometric properties of the Supin–Upper Tons watershed derived from the different datasets

Morphometric properties	SRTM DEM (90 m) (S90)		ASTER GDEM (30 m) (A30)		Cartosat-1 DEM (30 m) (C30)		Topographical map (1:250,000)		Topographical map (1:50,000)			ASTER GDEM (resampled 90 m) (A90)	Cartosat-1 DEM (resampled 90 m) (C90)
									Surveyed (T50A)	DEM derived (T50D)		Overall	Overall
	Overall	Ice free area	Overall	Ice free area	Overall	Ice free area	Surveyed (T250A)	DEM derived (T250D)	–	Overall	Ice free area	–	–
*Catchment size properties*													
Basin area (sq km)	976.20	498.00	977.40	498.90	970.80	497.40	955.22	958.80	978.15	973.40	831.40	1197	1188
Basin perimeter (km)	214.90	266.40	179.00	255.70	188.50	258.20	164.91	164.40	171.02	169.20	170.45	188.7	191.9
Basin length (km)	52.63		52.85		52.50		51.33	51.64	52.80	52.65		54.3	53.5
Main channel length (km)	57.86	47.60	60.37	48.54	60.38	48.34	49.76	62.91	47.21	62.54	47.18	73.35	70.27
Total channel length (km)	1984.12	861.82	3760.92	1822.78	3921.75	1868.98	493.67	1434.91	1733.43	1474.66	619.84	2264.57	2333.84
Total number of stream segments	2457	1036	8314	4141	7832	3717	132	1376	2060	1379	575	2542	2467
Stream order	6	6	7	7	7	6	4	6	6	6	5	6	6
*Relief properties*													
Absolute relief (m)—maximum	6307		6284		5891		6351		6317			6254	5882
Absolute relief (m)—minimum	1275		1282		1229		1343		1302			1306	1248
Mean relief (m)	3742.05		3939.30		3875.07		3762.95		3931.33			3929.65	3864.58
Relative relief (m)	5032		5002		4662		5008		5015			4948	4634
Relief ratio	95.61		94.65		88.80		97.56		95.61			91.12	86.62
Hypsometric integral	0.53		0.53		0.56		0.49		0.52			0.53	0.56
*Shape properties*													
Circularity ratio	0.27		0.38		0.34		0.44		0.27			0.42	0.41
Elongation ratio	0.67		0.67		0.67		0.68		0.67			0.72	0.73
*Texture properties*													
Drainage density (sq km/ km)	2.03	1.73	3.85	3.65	4.04	3.76	0.52	1.50	1.77	1.51	0.75	1.89	1.96
Constant of channel maintenance (sq km/km)	0.49	0.58	0.26	0.27	0.25	0.27	1.93	0.67	0.56	0.66	1.34	0.53	0.51
Stream frequency (no. per sq km)	2.52	2.08	8.51	8.30	8.07	7.47	0.14	1.44	2.11	1.42	0.69	2.12	2.08
Bifurcation ratio	4.58	4.05	4.41	4.06	4.31	5.29	4.85	4.22	4.44	4.24	5.29	4.72	4.56

As expected, DEM resolution and map scale are significant factors in determining the various morphometric attributes. There is a slight variation in the extent of basin areas extracted from different DEMs, but no marked changes in basin shape parameters arise. The basins derived are all of similar shape and preserve their aspect ratio regardless of DEM resolution or map scale. While extracting Basin Elevation parameters, the data derived from the Cartosat-1 DEM datasets (30 m and resampled 90 m) show the greatest difference from the other datasets in a range of 300 m or more. For example, it is evident from Table 2 that the maximum height in the drainage basin ranges from 6254 m to 6351 m for the DEMs obtained from SRTM, ASTER and topographical maps which yields a range of only 100 m. But the maximum elevation for the Cartosat-1 DEM is 5891 m for the 30 m resolution dataset and 5882 m for the Cartosat-1 DEM resampled at 90 m resolution. This implies that the extreme values in the Cartosat-1 DEM dataset vary markedly, relative to the other databases, especially for this study area. It can be clearly observed that the basin areas obtained from SRTM 90 m, ASTER GDEM 30 m, and Cartosat-1 DEM 30 m show the highest degree of correlation. Other DEMs, especially the 90 m resampled DEMs of ASTER and Cartosat-1 data, also show wide fluctuations.

**Fig. 10:**
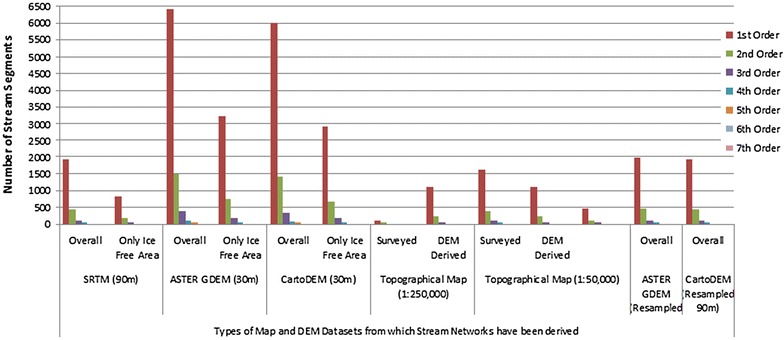
Comparison of stream counts after Strahler (1954). Order-wise stream networks have been derived from different datasets

**Fig. 11:**
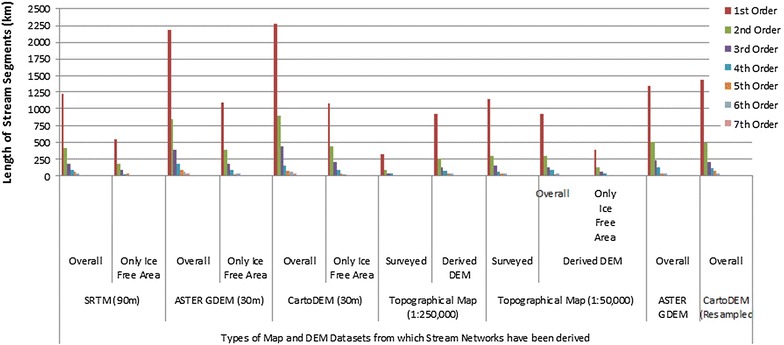
Comparison of total stream lengths after Strahler (1954). Order-wise stream networks have been derived from different datasets

 Figures 10 and 11 depict the comparisons for DEMs derived from the different datasets with respect to stream counts and total stream lengths respectively. It is observed that the maximum number of streams are generated by the two 30 m DEM datasets, namely the ASTER and Cartosat-1. Moreover, the highest order of 7 (following the Strahler Stream Segment Ordering Scheme—Strahler 1954) is also depicted by these two DEM datasets. Most of the other DEM datasets give a highest order of 6. The DEMs derived from topographical maps show the coarsest resolution i.e. highest stream order of 4.

In case of stream lengths, it is again observed that the overall stream lengths are highest for the two finer resolutions DEMs i.e. ASTER GDEM 30 m and the Cartosat-1 DEM 30 m. In contrast the DEMs obtained from the topographical maps actually report both, a lower number as well as a lesser length of streams, than the actual surveyed topographical map streams.

Drainage densities derived from the 30 m DEM data are much higher than those from lesser DEM resolution and map counterparts. Drainage texture parameters and bifurcation ratio are similar across the board between SRTM 90 m, ASTER 90 m, Cartosat-1 90 m DEMs and topographical map derived networks. In case of drainage density, it is observed that the results obtained from the 30 m DEM (ASTER and Cartosat 1) are the highest, ranging from 3.65 km/sq km for the ice free area of ASTER GDEM 30 m and 4.04 km/sq km for Cartsoat-1 DEM of 30 m resolution. In case of topographical maps, for the US AMS map at 1:250,000 scale, the drainage density obtained for the DEM derived network (1.50 km/sq km) is much higher than the surveyed network in the map of the same scale (0.52 km/sq km), possibly indicative of the cartographic representation limitations in the latter. The 1:50,000 scale maps prepared by the SoI however reveal more comparable results between the topomap derived DEM network and actual surveyed network, wherein the larger map scale has feasibly allowed a more detailed stream network delineation. Similar results were obtained for the stream frequency data. Bifurcation ratios are quite similar throughout the datasets.

The difference or deviation of each of the above attributes have been computed for all the datasets, keeping the values extracted for these parameters from the 1:50,000 scale topographical map as constant, for comparison of the degree of change. The percentage change of this is presented (Table 3), which reinforces the discussion above. Across the different datasets, for almost all the parameters, the percentage difference is the least in case of the ASTER 30 m DEM surface derived values, thus attesting to its validity for obtaining morphometric values closest to those of a surveyed network.

**Table 3:** Computed deviations (in %) of the respective parameter-wise values for each dataset from those of the surveyed toposheet values

Morphometric parameters	V-T50A	Amount of deviation of parameter values of respective datasets from T50A values (%)						
		SRTM DEM (90 m) (S90)	ASTER GDEM (30 m) (A30)	Cartosat-1 DEM (30 m) (C30)	Topographical map (1:250,000) (T250A)	Topographical map (1:50,000) (T50A)	ASTER GDEM (resampled 90 m) (A90)	Cartosat-1 DEM (resampled 90 m) (C90)
*Catchment size properties*								
Basin Area (sq km)	973.40	+0.29	+0.41	−0.27	−1.87	0	+22.97	+22.05
Basin perimeter (km)	169.20	+27.01	+5.79	+11.41	−2.54	0	+11.52	+13.42
Basin length (km)	52.65	−0.04	+0.38	−0.28	−1.92	0	+3.13	+1.61
Main channel length (m)	62.54	−7.48	−3.47	−3.45	+0.59	0	+17.29	+12.36
Total channel length (km)	1733.40	+14.46	+116.96	+126.24	−17.22	0	+30.64	+34.64
Total no. of stream segments	1379	+78.17	+502.90	+462.95	−0.22	0	+84.34	+78.90
*Relief attributes*								
Maximum relief (m)	6317	−0.16	−0.52	−6.74	+0.54	0	−1.00	−6.89
Minimum relief (m)	1302	−2.07	−1.54	−5.61	+3.15	0	+0.31	−4.15
Mean relief (m)	3931.33	−4.81	+0.20	−1.43	−4.28	0	−0.04	−1.70
Relative relief (m)	5015	+0.34	−0.26	−7.04	−0.14	0	−1.34	−7.60
Relief ratio	95.61	0	−1.00	−7.12	+2.04	0	−4.70	−9.40
Hypsometric integral	0.52	+1.92	+1.92	+7.69	−5.77	0	+1.92	+7.69
*Surface attributes*								
Maximum slope (degrees)	69.25	+5.04	+20.95	+19.99	−11.58	0	+1.17	−5.40
Mean slope (degrees)	26.24	+8.003	+14.558	+11.17	−22.98	0	−5.72	−14.02
*Shape properties*								
Circularity ratio	0.27	0	+40.74	+25.23	+62.96	0	+55.56	+51.83
Elongation ratio	0.67	0	0	0	+1.49	0	+7.46	+8.96
*Drainage textural parameters*								
Bifurcation ratio	+4.24	+8.02	+4.01	+1.65	+14.38	0	+11.32	+7.54

*V-T50A* values from the DEM derived from topographical maps of scale 1:50,000

### Variations in the river longitudinal profiles

**Fig. 12:**
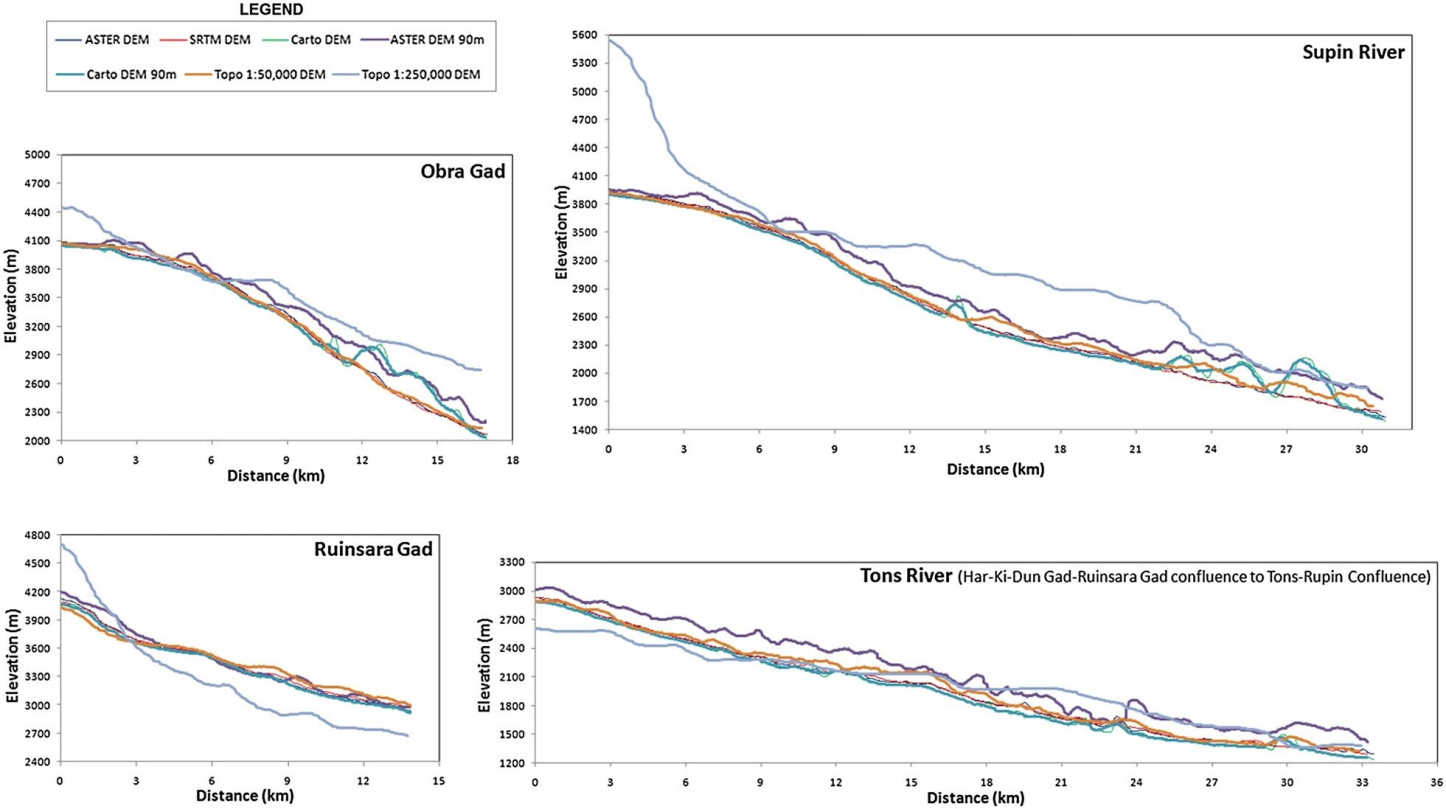
Longitudinal profiles of major streams of the Supin–Upper Tons Watershed obtained from different DEMs. Within the Supin–Upper Tons basin, Obra Gad and Ruinsara Gad respectively act as the principal tributaries of the Supin and Tons River, prior to the Supin–Tons confluence. The longitudinal profile of Tons River covers the stretch between the Har-Ki-Dun Gad–Ruinsara Gad confluence till the Tons–Rupin confluence (outlet of the basin)

River longitudinal profiles are used as an important parameter in geomorphometry and tectonic geomorphology (Lee and Tsai 2009). Therefore, the river longitudinal profiles of the Supin–Upper Tons and its tributaries have been taken as a parameter for investigating of the reliability of the various DEMs. Figure 12 depicts the longitudinal profiles derived from different DEMs along the Obra Gad, Supin River, Ruinsara Gad and Tons River respectively. The notable point that emerged from the profile plot and its form analysis is that although the graphs derived from the SRTM DEM 90 m, ASTER-GDEM 30 m and Cartosat-1 DEM 30 m datasets correlate quite closely, yet the Cartosat-1 data shows artifacts which causes spikes in the profile and therefore loses its reliability to a considerable extent. It may be mentioned here that the spikes in the Cartosat-1 data remain intact, in spite of the 11 pixel moving average method employed for smoothening the data. These spikes are also observed in the longitudinal profiles obtained from the resampled and coarsened DEMs of ASTER and CartoDEM at 90 m and therefore, do not fulfill the need. Highest amount of spiking and artifacts are found in the DEM derived from the topographical maps especially for the USAMS dataset at 1:250,000 scale. However, the DEM obtained from the SoI topographical sheets at 1:50,000 scale presents longitudinal profiles for these different rivers which is quite comparable with the profiles obtained from other DEMs, due to its inherent larger scale representation.

### Differences in waypoint elevations

**Fig. 13:**
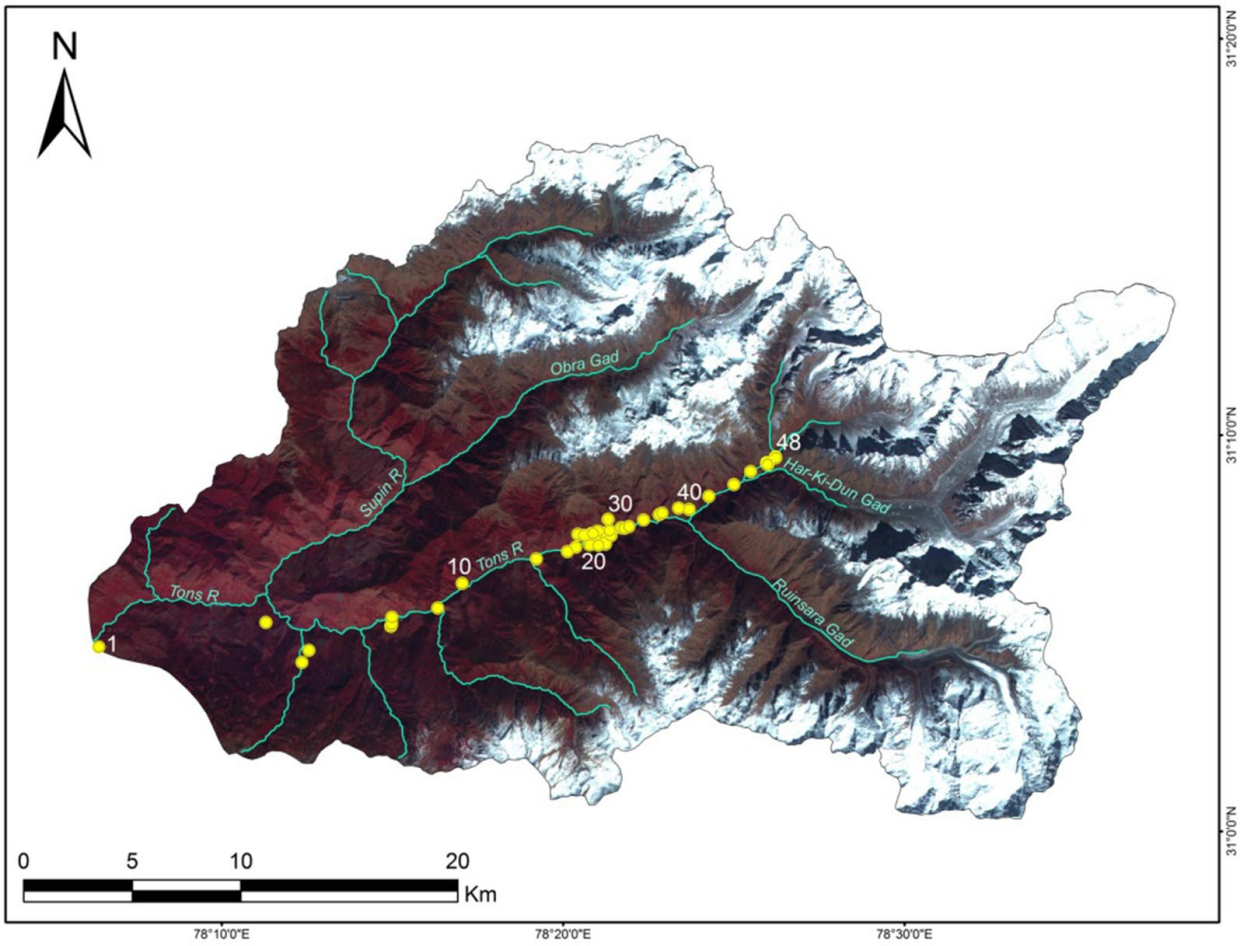
Distribution of GPS readings within the Supin–Upper Tons basin superposed on an October 2008 IRS LISS-3 image. The waypoints have largely been taken in the non-vegetative areas of the Tons valley, which is the most accessible part of the basin

**Fig. 14:**
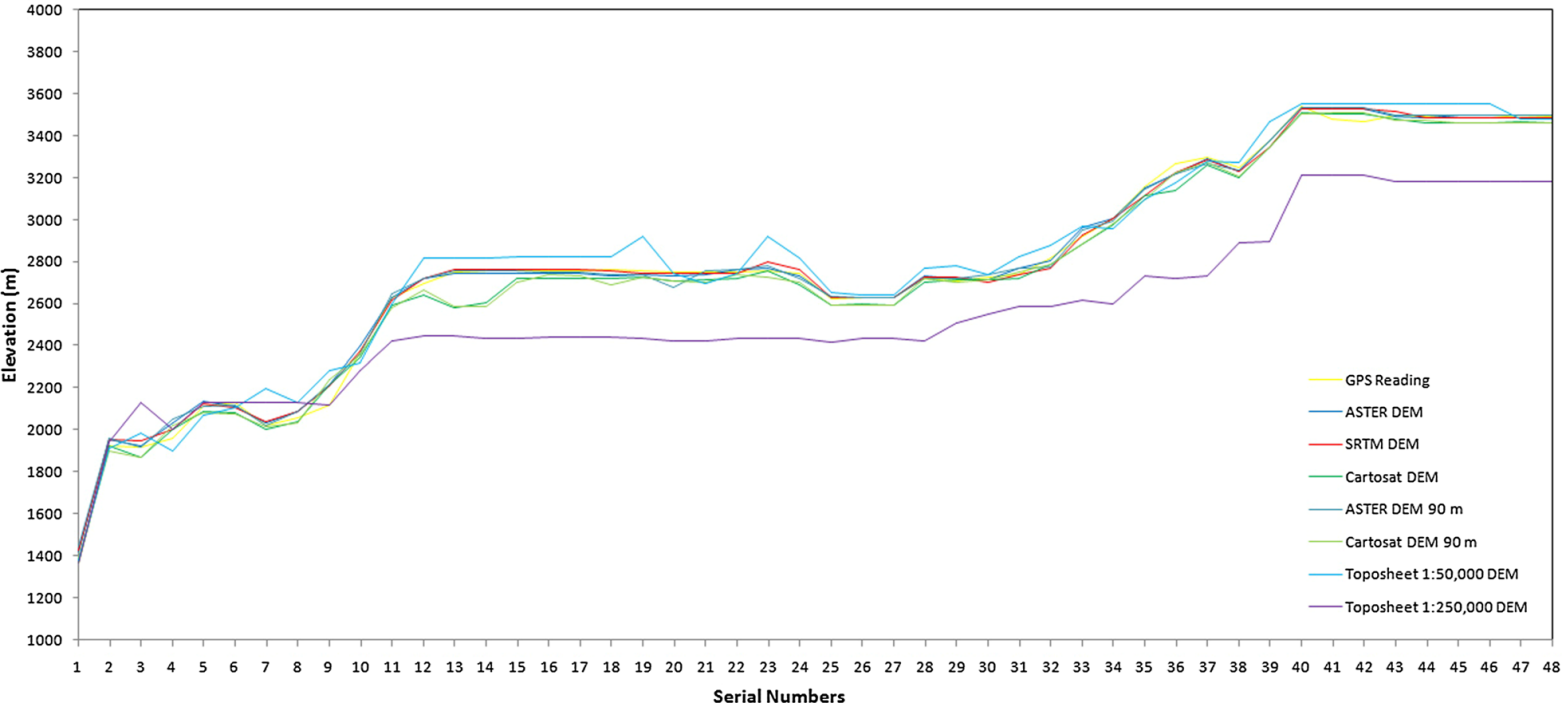
Differences in elevation data: GPS readings at different waypoints and different DEMs

**Fig. 15:**
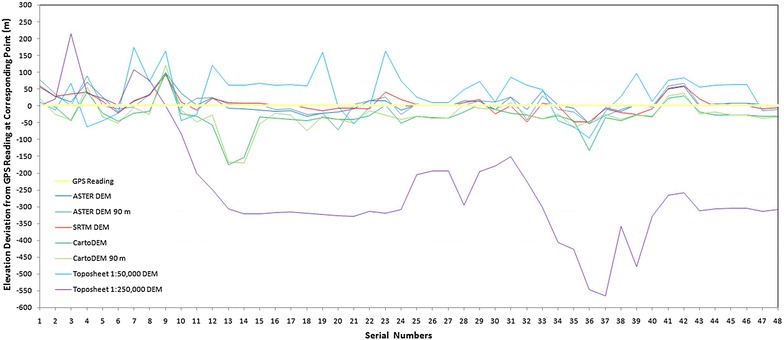
Elevation deviations of different DEMs from GPS readings at different waypoints

It is evident that the datasets, on basis of their differing resolutions, show subtle variations amongst each other when it comes to the longitudinal profiles. However, the relief and texture attributes of the drainage basin are found to be least sensitive to the dataset from which they are derived. In order to identify the most accurate DEM dataset in terms of measured elevation, some waypoint elevations have been taken into account. Global Positioning System (GPS) readings have been taken along different waypoints in the accessible parts of the Supin–Upper Tons basin in a reconnaissance field survey carried out in the area in April 2012 (Fig. 13). It is pertinent to mention here that the GPS readings have been taken by setting the instrument on the WGS 84 ellipsoid and that all the data depicted, were also converted to the same datum. This was essential in order to remove any sort of errors that might have been incurred due to datum and ellipsoid conflicts among the different data sources. The GPS waypoints were collected in open, level tracts within the basin, devoid of overhanging or nearby tree cover, to minimise any multi-path effects on the signal. Presumed to be the most accurate, these elevation values obtained via this GPS Survey have been compared with the elevations of the same points in the different DEMs and the differences in altitude between the GPS readings and the DEM readings were computed (Figs. 14, 15). The results have been presented in Table 4. It is evident that the SRTM-DEM gives the most reliable results when compared with the GPS readings, while next in reliability is the ASTER GDEM 30 m dataset.

**Table 4:** Deviations of elevation data of different digital elevation models from collected global positioning system survey data

	SRTM DEM (90 m) (S90)	ASTER GDEM (30 m) (A30)	Cartosat-1 DEM (30 m) (C30)	ASTER GDEM (resampled 90 m) (A90)	Cartosat-1 DEM (resampled 90 m) (C90)	Topographical map (1:250,000) (T250A)	Topographical map (1:50,000) (T50A)
Standard deviation (SD)	27.15	27.61	41.95	32.11	43.95	167.07	62.58
Mean deviation (MD)	19.68	20.51	23.08	21.86	24.79	128.80	50.91

The indigenous Cartosat-1 DEM shows marked deviations from the GPS readings which put a question mark on its accuracy in this case. The DEM derived from the topographical map of scale 1:250,000 shows the highest amount of deviation from the GPS readings (Std. Dev. 167). The DEM derived from the SoI map of 1:50,000 scale is more reliable but in comparison to the DEMs obtained from digital sources like SRTM, ASTER and Cartosat-1, its reliability is low (SD 63). Therefore, it may be surmised with a fair degree of certainty that some of these readily available DEMs could be far more reliable options for morphometric analysis compared to the traditional smaller-scale topographical maps, especially in such rugged terrain. Among the DEMs, the accuracy and reliability of the SRTM DEM and ASTER GDEM exceeds than that of the indigenous Cartosat-1 DEM.

### Variations in morphometric parameters extracted grid-wise

Maps of four primary morphometric parameters have been prepared after extracting their respective values for each of the 1154 grids overlain across the corresponding basin surfaces-average elevation, relative relief, slope and drainage density.

**Fig. 16:**
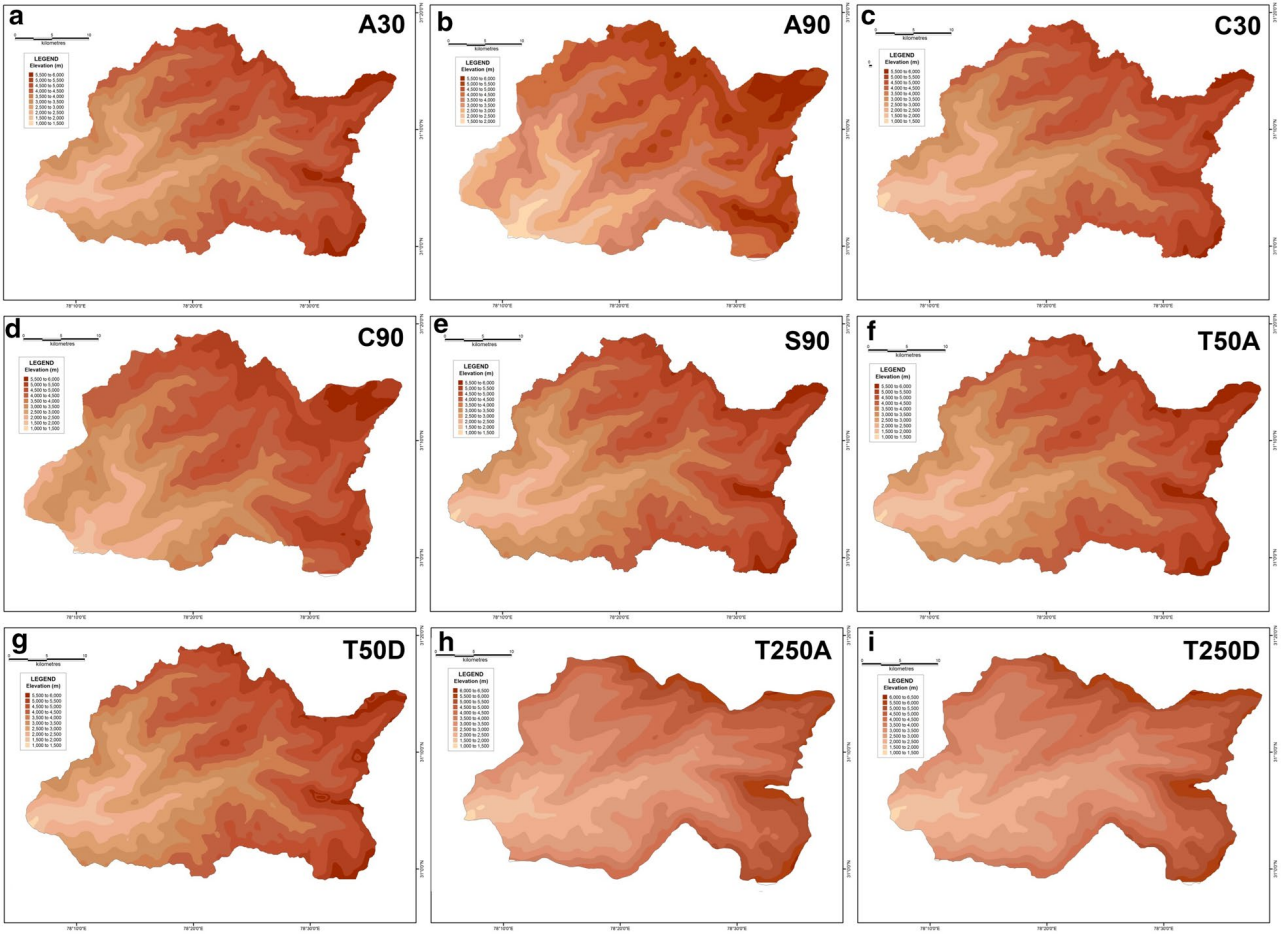
Mean elevation zone maps prepared for the different datasets on basis of extracted grid-wise values. ASTER 30 m (**a**), Resampled ASTER 90 m (**b**), CartoDEM 30 m (**c**), Resampled CartoDEM 90 m (**d**), SRTM 90 m (**e**), 1:50,000 SoI toposheet (**f**), Resampled 90 m DEM from 1:50,000 SoI toposheet (**g**), 1:250,000 USAMS toposheet (**h**), Resampled 90 m DEM from 1:250,000 USAMS toposheet (**i**)

**Fig. 17:**
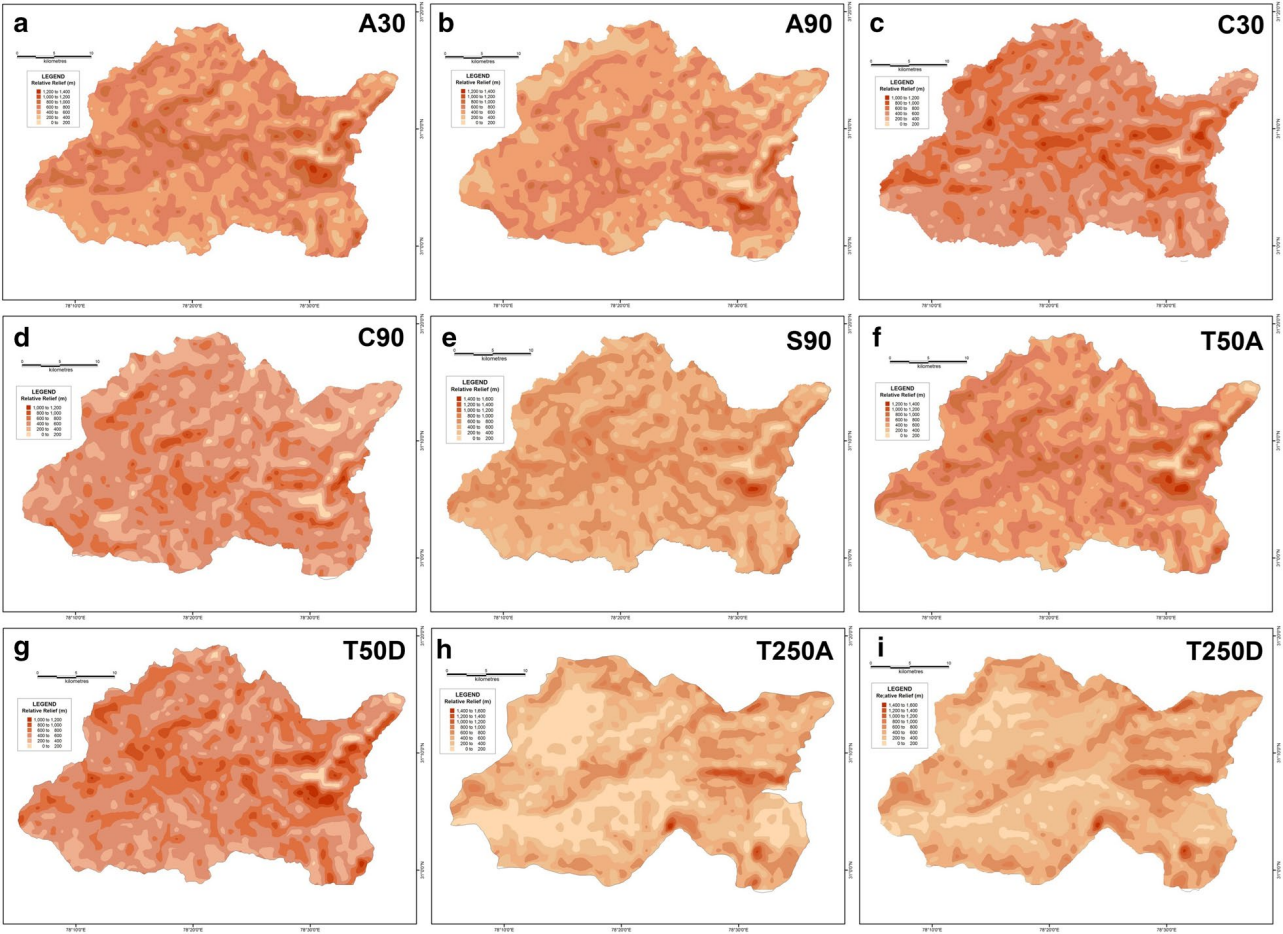
Relative relief zone maps prepared for the different datasets on basis of extracted grid-wise values. ASTER 30 m (**a**), resampled ASTER 90 m (**b**), CartoDEM 30 m (**c**), resampled CartoDEM 90 m (**d**), SRTM 90 m (**e**), 1:50,000 SoI toposheet (**f**), resampled 90 m DEM from 1:50,000 SoI toposheet (**g**), 1:250,000 USAMS toposheet (**h**), resampled 90 m DEM from 1:250,000 USAMS toposheet (**i**)

**Fig. 18:**
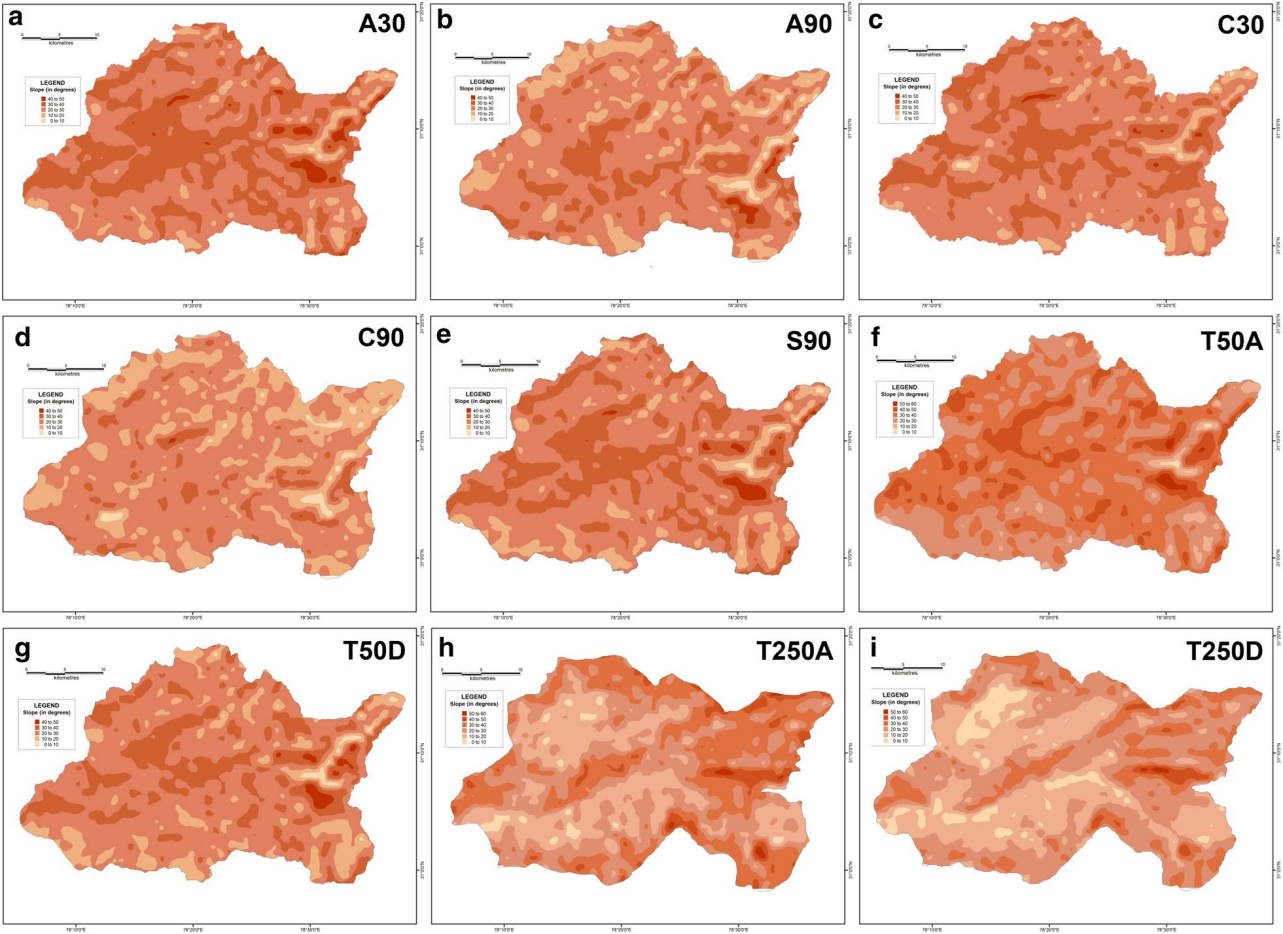
Slope zone maps prepared for the different datasets on basis of extracted grid-wise values. ASTER 30 m (**a**), resampled ASTER 90 m (**b**), CartoDEM 30 m (**c**), resampled CartoDEM 90 m (**d**), SRTM 90 m (**e**), 1:50,000 SoI toposheet (**f**), resampled 90 m DEM from 1:50,000 SoI toposheet (**g**), 1:250,000 USAMS toposheet (**h**), resampled 90 m DEM from 1:250,000 USAMS toposheet (**i**)

**Fig. 19:**
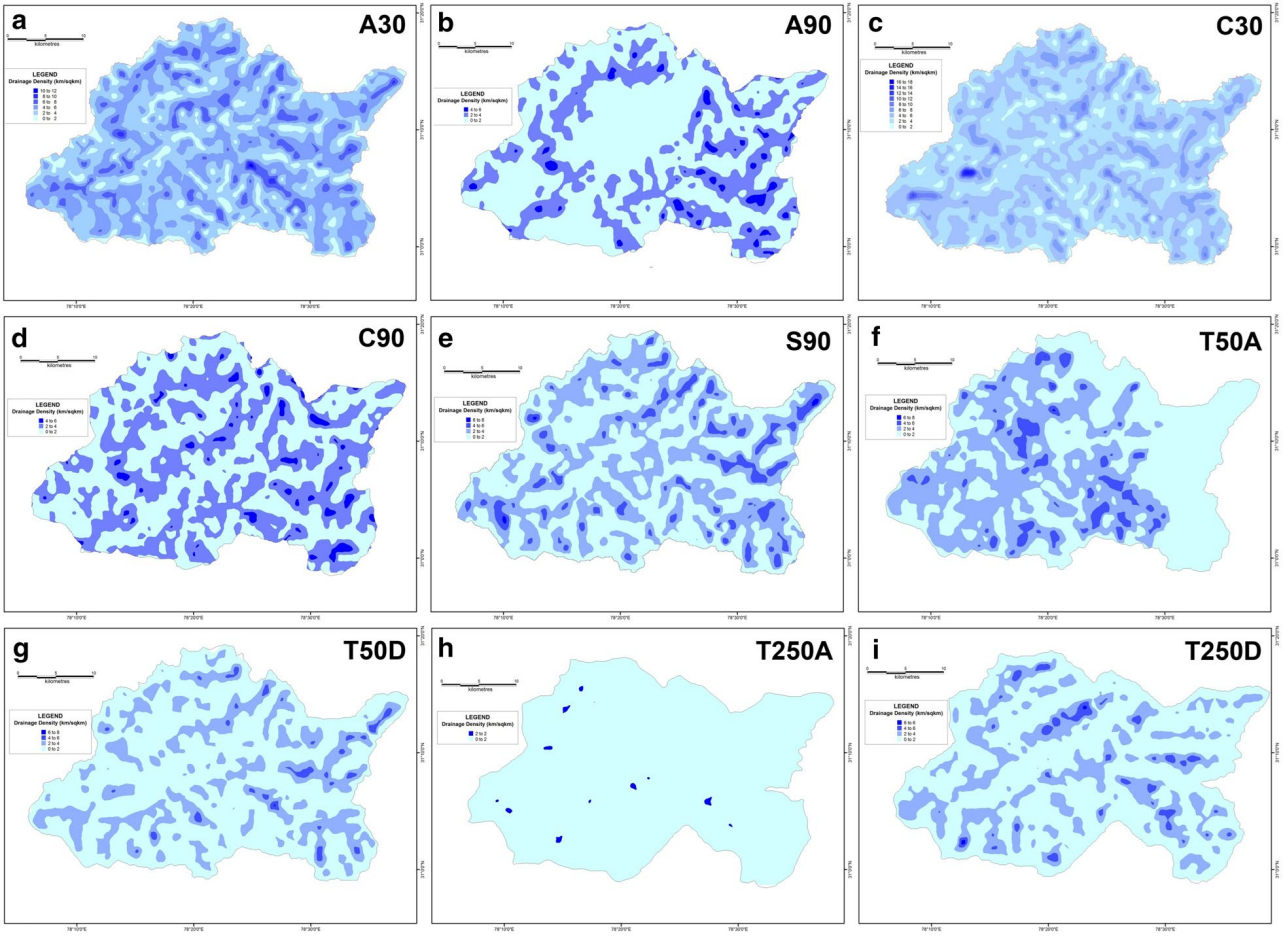
Drainage density zone maps prepared for the different datasets on basis of extracted grid-wise values. ASTER 30 m (**a**), resampled ASTER 90 m (**b**), CartoDEM 30 m (**c**), Resampled CartoDEM 90 m (**d**), SRTM 90 m (**e**), 1:50,000 SoI toposheet (**f**), resampled 90 m DEM from 1:50,000 SoI toposheet (**g**), 1:250,000 USAMS toposheet (**h**), resampled 90 m DEM from 1:250,000 USAMS toposheet (**i**)

From the mean elevation maps generated from the various DEM-derived datasets, it is revealed that the average elevation increases from west to east for the Supin–Upper Tons watershed (Fig. 16), and ranges from 1000 m to 6500 m above mean sea level. Furthermore, the results obtained for the different datasets do not match each other. For example, the SRTM 90 m, ASTER 30 m and CartoDEM 30 m datasets display comparable results with the 1:50,000 topographical maps and portray the divides and valleys quite prominently, while the 1:250,000 topographical map and the DEM derived from it and the other resampled 90 m DEMs from ASTER and CartoDEM deviate markedly from the general trend. This is the expected outcome given their coarser resolution. The respective relative relief maps (Fig. 17) and slope maps (Fig. 18), further show the utility of these finer resolution DEMs (especially the ASTER 30 m DEM) in extracting these values and adequately representing actual terrain features. The ASTER 30 m shows the intervening ridge along the central part of the basin, between the Tons and Supin valleys very clearly (with high relief and slope values) and also highlights the presence of these two valleys on either side of it. The other DEM datasets fail to do so as clearly, with the DEM prepared from the coarsest resolution 1:250,000 USAMS topographical map, being particularly unrepresentative of the various terrain features. The higher resolution DEMs allow more extensive drainage network extraction and thus log higher drainage density values, particular along valley floor flow convergence zones (Fig. 19). While the overall drainage network can be discerned clearly from their isopleth maps, the coarser resolution datasets fail to represent similar attributes with such clarity.

While, the above isopleth maps allow visual comparison, a deeper statistical insight has been sought into the grid-wise variations that have occurred in extracting the same parameters from different map and DEM datasets. For this, the differences for each of these parameters from its corresponding value in the other datasets was computed grid-wise. It should be noted that the positive or negative sign for each value shall reverse if the position of the variables in the subtraction formulae are inverted. Their respective percentage of variation was noted accordingly and these were then averaged (Table 5). Where the datasets would match perfectly, this averaged value would approach zero. It is seen that in most cases, the ASTER 30 m dataset shows the least mean percentage difference for these four parameters, especially when compared to the SoI topographical map and DEM dataset, implying its close relation with the surveyed database. To do away with the possibility of positive and negative differences cancelling each other out and to better ferret out the true divergences among the parameter values that exists among these datasets, the coefficient of variation has been computed for the corresponding percentage differences in mean elevation (Table 6), relative relief (Table 7), slope (Table 8) and drainage density (Table 9). These tables again confirm that the ASTER 30 DEM dataset usually exhibits the least degree of variation for the above parameters.

**Table 5:** Averaged percentage difference between corresponding grid-wise morphometric values across the different datasets for four select parameters

Difference from	Difference computation	Averaged percentage difference			
		Mean elevation	Relative relief	Slope	Drainage density
A30	A30–A90	−4.11	5.07	7.92	34.27
	A30–C30	1.67	7.86	5.25	−11.69
	A30–C90	−2.40	14.50	16.65	31.13
	A30–S90	0.08	6.72	6.19	53.51
	A30–T50D	−0.05	13.05	11.47	69.78
	A30–T250D	2.74	14.58	15.47	63.24
	A30–T50A	−0.48	6.66	−9.14	54.85
	A30–T250A	2.69	27.77	1.12	87.56
A90	A90–C30	1.48	−19.07	−18.85	−504.00
	A90–C90	1.58	8.17	7.93	−70.47
	A90–S90	−0.12	−22.34	−19.48	−186.39
	A90–T50D	−0.24	−13.06	−12.25	−67.20
	A90–T250D	2.82	−4.72	−2.83	−98.14
	A90–T50A	−0.65	−21.70	−37.34	−124.36
	A90–T250A	2.77	10.51	−20.11	35.06
C30	C30–C90	−4.16	5.72	11.09	38.45
	C30–S90	−1.65	−4.84	−1.28	55.52
	C30–T50D	−1.77	2.64	4.57	71.10
	C30–T250D	1.10	5.63	9.75	65.97
	C30–T50A	−2.22	−4.72	−17.82	58.67
	C30–T250A	1.04	20.13	−5.61	88.51
C90	C90–S90	−1.71	−36.30	−31.12	−293.65
	C90–T50D	−1.83	−26.08	−23.10	−188.21
	C90–T250D	1.30	−17.21	−13.02	−76.93
	C90–T50A	−2.25	−35.80	−50.84	−116.31
	C90–T250A	1.24	−0.18	−31.80	30.39
S90	S90–T50D	−0.12	6.54	5.55	28.85
	S90–T250D	2.66	7.03	8.49	−72.73
	S90–T50A	−0.56	−0.48	−16.71	−71.97
	S90–T250A	2.61	21.32	−6.97	58.97
T50D	T50D–T250D	2.79	−5.73	0.17	−123.76
	T50D–T50A	−0.45	−7.67	−23.72	−159.61
	T50D–T250A	2.74	9.09	−16.37	31.33
T250D	T250D–T50A	−4.46	−88.98	−103.15	−187.97
	T250D–T250A	−0.05	−60.11	−24.68	56.10
T50A	T50A–T250A	3.12	11.96	−18.57	49.16

The difference between corresponding values of two parameters for each of the 1154 one sqkm grids overlain across the basin surface has been first computed. Then the % difference was computed, again for each of these grids, by dividing the computed difference for each by the corresponding parameter value from which the subtraction is done, multiplied by 100. The mean of these % difference values for the 1154 grids has then been tabulated here

**Table 6:** Coefficient of variation of the percentage difference in grid-wise mean elevation between the respective datasets

y	x								
	A30	A90	C30	C90	S90	T50D	T250D	T50A	T250A
A30	0								
A90	−5.42	0							
C30	0.97	13.74	0						
C90	−8.98	1.05	−5.29	0					
S90	7.08	−173.78	−1.14	−12.28	0				
T50D	−47.45	−86.19	−1.56	−11.49	−16.76	0			
T250D	3.83	7.57	9.57	16.69	3.95	3.69	0		
T50A	−5.86	−31.98	−1.48	−9.39	−4.99	−5.38	−2.48	0	
T250A	3.93	7.72	10.13	17.42	4.05	3.78	−22.24	3.39	0

The difference is computed as (x − y) for each of 1154 one sqkm grids across basin surface from respective values

Percentage Difference = ((x − y)/x) * 100; Lower positive or negative values imply a greater similarity between values of two datasets

**Table 7:** Coefficient of variation of the percentage difference in grid-wise relative relief between the respective datasets

y	x								
	A30	A90	C30	C90	S90	T50D	T250D	T50A	T250A
A30	0								
A90	8.94	0							
C30	1.99	−3.78	0						
C90	2.74	2.19	7.64	0					
S90	1.21	−3.25	−5.35	−2.20	0				
T50D	1.49	−5.38	11.20	−2.99	3.12	0			
T250D	3.94	−16.91	10.93	−5.19	9.57	−15.55	0		
T50A	3.51	−3.62	−7.34	−2.44	−53.49	−2.50	−7.99	0	
T250A	2.10	7.38	3.10	−485.15	3.17	10.61	−38.47	9.71	0

The difference is computed as (x − y) for each of 1154 one sqkm grids across basin surface from respective values

Percentage Difference = ((x − y)/x) * 100; Lower positive or negative values imply a greater similarity between values of two datasets

**Table 8:** Coefficient of Variation of the Percentage Difference in Grid-wise Slope between the respective datasets

y	x								
	A30	A90	C30	C90	S90	T50D	T250D	T50A	T250A
A30	0								
A90	4.41	0							
C30	2.23	−3.04	0						
C90	1.84	1.77	3.17	0					
S90	0.96	−3.07	−19.48	−2.07	0				
T50D	1.31	−4.71	5.98	−2.69	2.84	0			
T250D	3.03	−23.25	5.15	−5.48	6.72	398.69	0		
T50A	−2.88	−1.95	−2.23	−1.56	−1.75	−1.16	−6.50	0	
T250A	45.67	−3.66	−9.84	−2.51	−8.87	−4.40	−2.12	−14.88	0

The difference is computed as (x − y) for each of 1154 one sqkm grids across basin surface from respective values

Percentage Difference = ((x − y)/x) * 100; Lower positive or negative values imply a greater similarity between values of two datasets

**Table 9:** Coefficient of variation of the percentage difference in grid-wise drainage density between the respective datasets

y	x								
	A30	A90	C30	C90	S90	T50D	T250D	T50A	T250A
A30	0								
A90	1.97	0							
C30	−3.19	−6.49	0						
C90	2.21	−17.99	1.45	0					
S90	0.43	−6.08	0.42	−13.91	0				
T50D	0.35	−7.44	0.33	−17.79	3.08	0			
T250D	0.67	−8.16	0.59	−10.44	−21.15	−7.42	0		
T50A	0.92	−8.55	0.74	−9.49	−10.27	−6.79	−7.46	0	
T250A	0.21	7.22	0.18	15.28	2.20	11.19	1.45	2.06	0

The difference is computed as (x − y) for each of 1154 one sqkm grids across basin surface from respective values

Percentage Difference = ((x − y)/x) * 100; Lower positive or negative values imply a greater similarity between values of two datasets

**Fig. 20:**
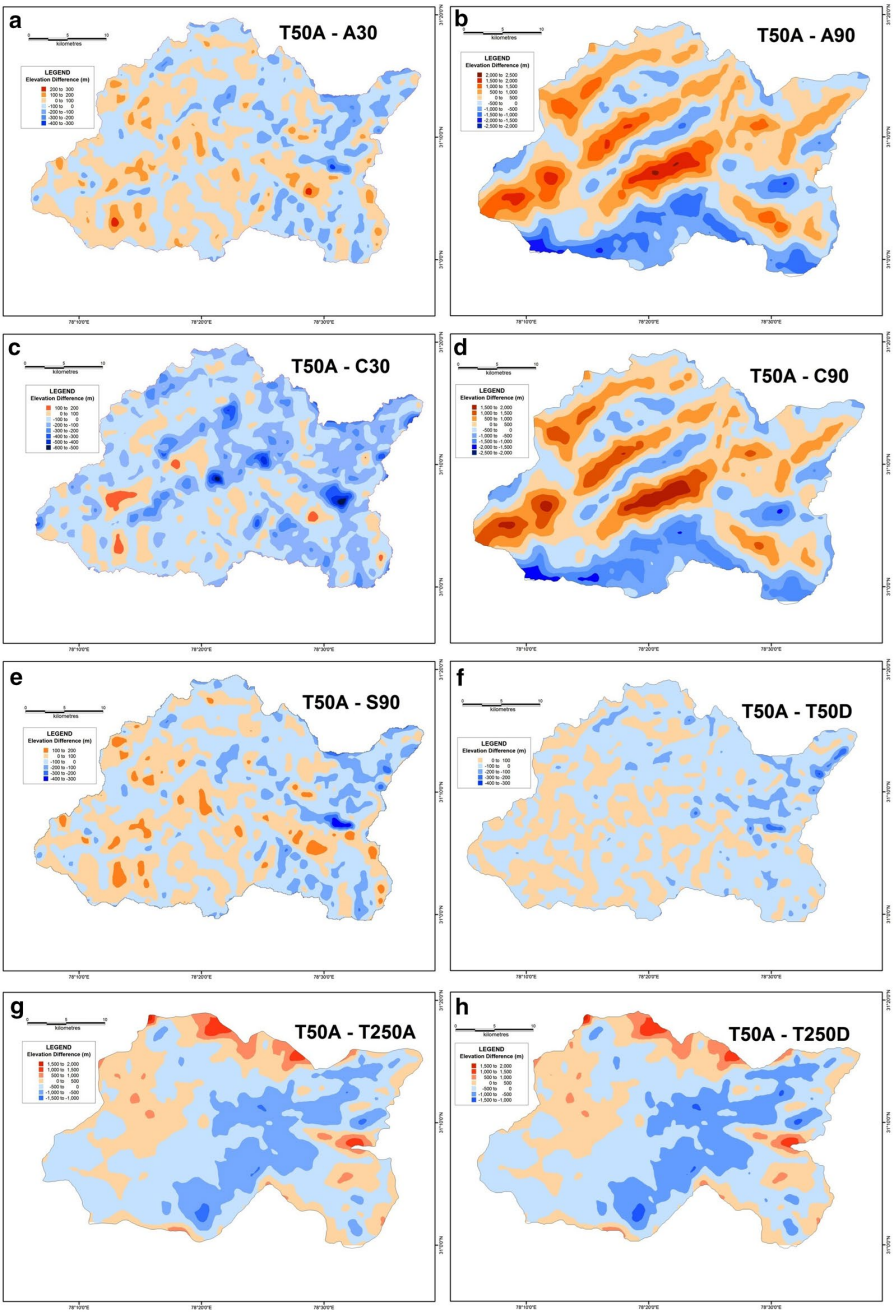
Isopleth zones prepared on basis of grid-wise difference of each dataset’s mean elevation values from the corresponding values of the SoI 1:50,000 topographical map. Relatively lesser positive (T50A values are higher) or negative (T50A values are lower) difference ranges imply a higher match with the topographical map values. Difference with ASTER 30 m (**a**), difference with resampled ASTER 90 m (**b**), difference with CartoDEM 30 m (**c**), difference with resampled CartoDEM 90 m (**d**), difference with SRTM 90 m (**e**), difference with resampled 90 m DEM from 1:50,000 SoI toposheet (**f**), difference with 1:250,000 USAMS toposheet (**g**), difference with resampled 90 m DEM from 1:250,000 USAMS elevation zone maps (**h**)

**Fig. 21:**
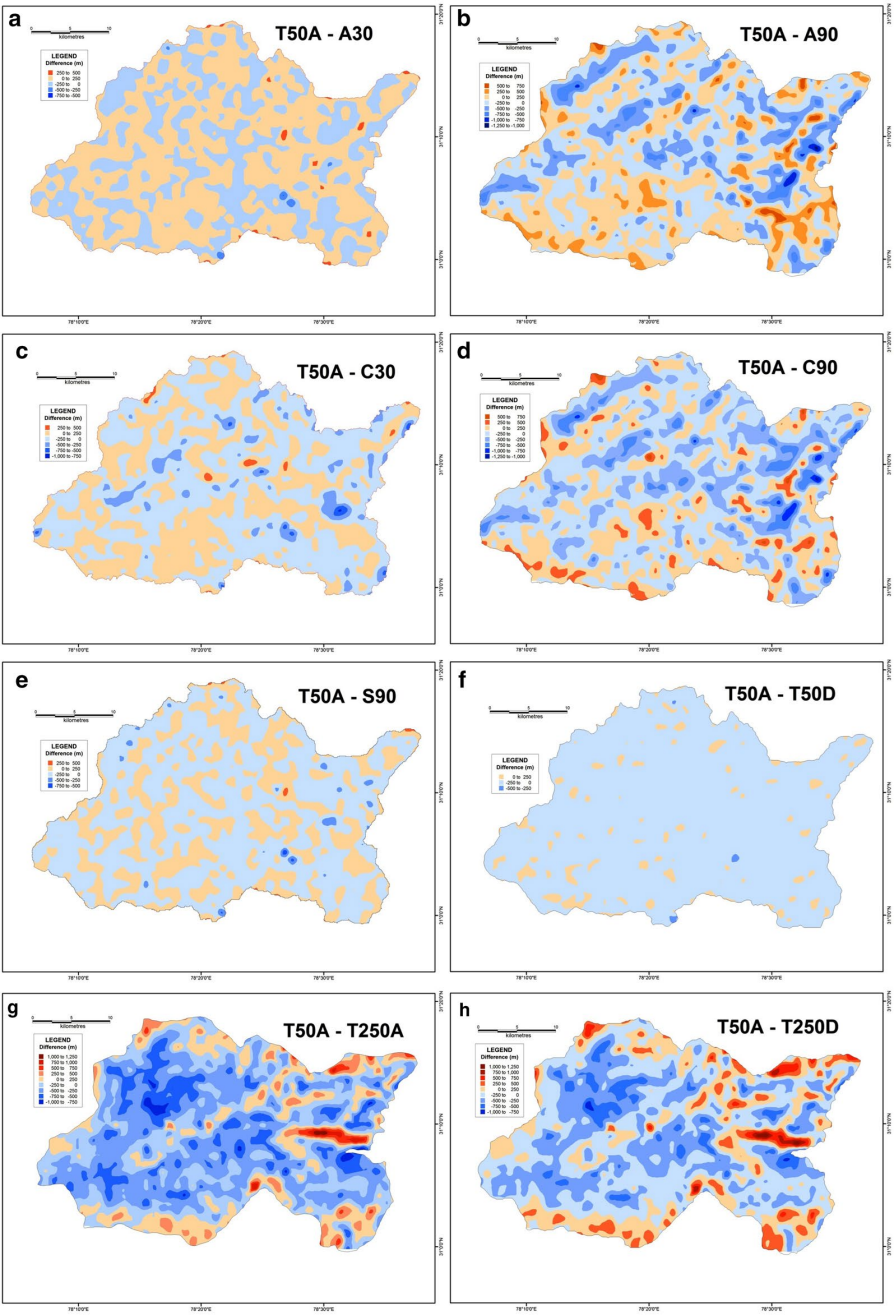
Isopleth zones prepared on basis of grid-wise difference of each dataset’s relative relief values from the corresponding values of the SoI 1:50,000 topographical map. Relatively lesser positive (T50A values are higher) or negative (T50A values are lower) difference ranges imply a higher match with the topographical map values. Difference with ASTER 30 m (**a**), difference with resampled ASTER 90 m (**b**), difference with CartoDEM 30 m (**c**), difference with resampled CartoDEM 90 m (**d**), difference with SRTM 90 m (**e**), difference with resampled 90 m DEM from 1:50,000 SoI toposheet (**f**), difference with 1:250,000 USAMS toposheet (**g**), difference with resampled 90 m DEM from 1:250,000 USAMS toposheet (**h**)

**Fig. 22:**
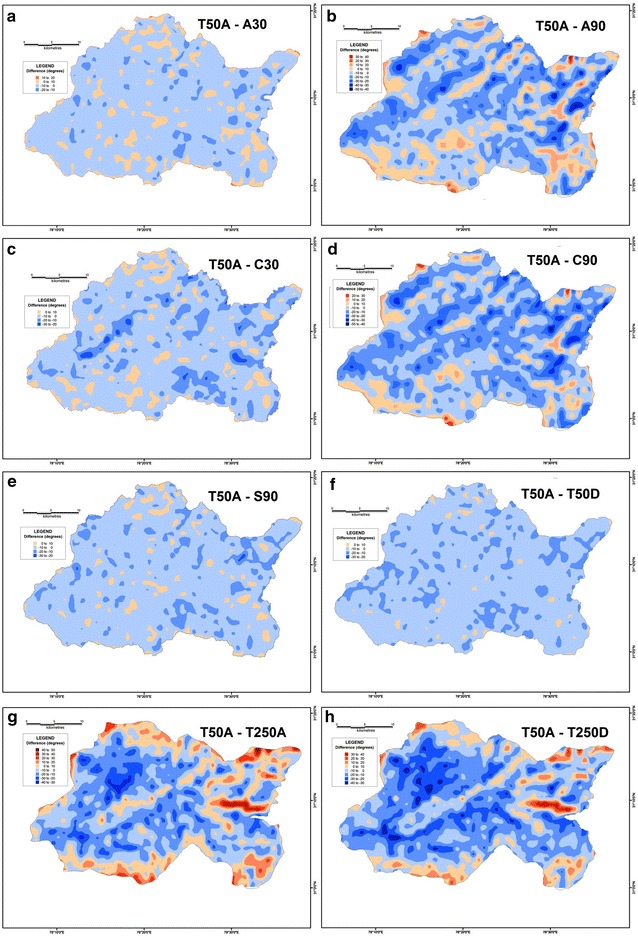
Isopleth zones prepared on basis of grid-wise difference of each dataset’s slope values from the corresponding values of the SoI 1:50,000 topographical map. Relatively lesser positive (T50A values are higher) or negative (T50A values are lower) difference ranges imply a higher match with the topographical map values. Difference with ASTER 30 m (**a**), difference with resampled ASTER 90 m (**b**), difference with CartoDEM 30 m (**c**), difference with resampled CartoDEM 90 m (**d**), difference with SRTM 90 m (**e**), difference with resampled 90 m DEM from 1:50,000 SoI toposheet (**f**), difference with 1:250,000 USAMS toposheet (**g**), difference with resampled 90 m DEM from 1:250,000 USAMS toposheet (**h**)

**Fig. 23:**
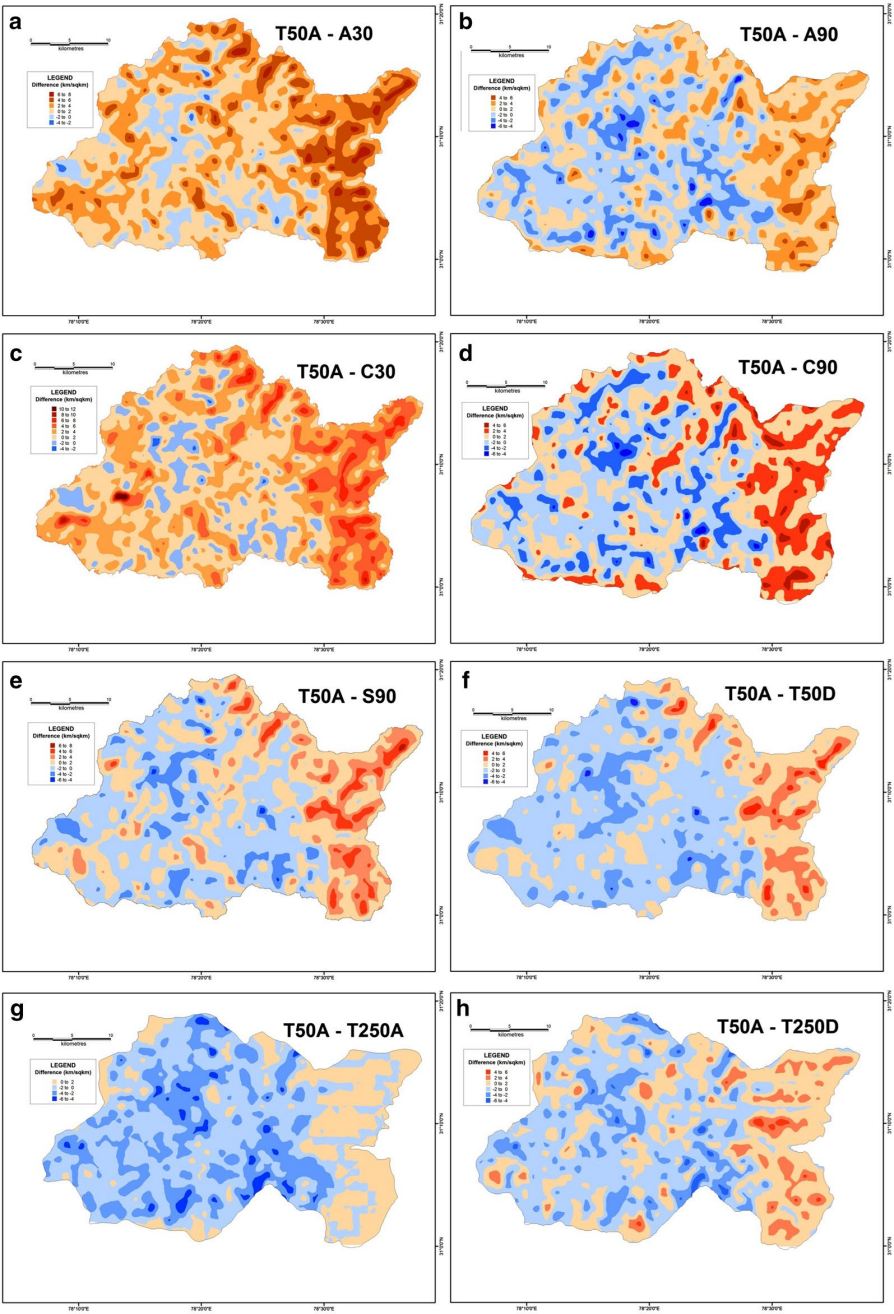
Isopleth zones prepared on basis of grid-wise difference of each dataset’s drainage density values from the corresponding values of the SoI 1:50,000 topographical map. Relatively lesser positive (T50A values are higher) or negative (T50A values are lower) difference ranges imply a higher match with the topographical map values. Difference with ASTER 30 m (**a**), difference with resampled ASTER 90 m (**b**), difference with CartoDEM 30 m (**c**), difference with resampled CartoDEM 90 m (**d**), difference with SRTM 90 m (**e**), difference with resampled 90 m DEM from 1:50,000 SoI toposheet (**f**), difference with 1:250,000 USAMS toposheet (**g**), difference with resampled 90 m DEM from 1:250,000 USAMS toposheet (**h**)

In order to determine the validity and reliability of any DEM, it is essential to compare their derived values against a reference frame through which their reliability can be gauged. The SoI topographical maps of R.F. 1:50,000 is taken to be the most suitable frame of reference since it is a large-scale surveyed database. Therefore, the results of different datasets have been compared with the corresponding values extracted from the 1:50,000 topographical maps in order to assess their reliability. In case of average elevation, the 30 m ASTER DEM along with the 90 m SRTM dataset have emerged as the most reliable. In both these datasets, the difference in elevation is within a range of 0–100 m, with a maximum of 400 m in isolated pockets (Fig. 20). However, the CartoDEM dataset does not appear to be very reliable as the values of its difference are much higher. The limitation of the CartoDEM is more pronounced near the downstream reach of the river i.e. in the areas of relatively lower elevation. Similar results were obtained when comparing the difference values obtained for the relative relief parameter, following the same procedure (Fig. 21), as well as for the difference maps prepared on basis of slope values (Fig. 22), wherein the ASTER 30 m dataset matches that from the 1:50,000 topographical map quite closely. The results obtained for the drainage density difference datasets are slightly different (Fig. 23). The ASTER and SRTM DEM datasets match the spatial pattern of the difference from the 1:50,000 topographical maps. Both these datasets reveal higher drainage densities in the lower reaches of the basin as compared to the topographical maps, and also in the upper basin reaches, since the streamlines can be extended further and extracted in greater detail from DEMs, than is demarcated in paper maps due to cartographical constraints. However, the results of the ASTER dataset appear to be more reliable than the SRTM dataset, especially in case of higher elevation areas. It is pertinent here to mention  that a previous study conducted by Hayakawa et al. (2008) had pointed out that the pre-release version of 30 m ASTER GDEM was shown to be superior to the 90 m SRTM DEM in Japan. This study corroborates those findings. The indigenous CartoDEM data (30 m resolution) is seemingly inferior to the existing global DEMs.

Again, to examine statistically this pattern of difference between the topographical map values for each parameter and its extracted values from the other DEM and map datasets, the proportionate basin area lying within the lowest positive and negative difference zones have been summed (Table 10). If their summation occupies almost the whole or a majority of the basin space (i.e. the closer this summation is to 100 %), then it would imply that the values of this particular dataset are quite close and approximate to those derived from the surveyed large-scale topographical map, and could thus be taken to represent reality more accurately, compared to the other datasets. This summation has been found out for each of the datasets for every parameter initially, and then averaged to show the final value across all four parameters, on which basis three groups have been prepared (Low match: Below 70 %; Moderate match: 70–80 % and High match: Above 80 %). It is clearly evident that values from the coarsest dataset (i.e., the 1:250,000 scale USAMS topographical map—T250A and T250D) have the lowest match, while, the resampled DEM (T50D) prepared from the larger scale SoI 1:50,000 scale topographical map, obviously match its parent database (T50A), the most. Among the other DEM datasets, both the ASTER 30 m and SRTM 90 m show very high matches but the CartoDEM 30 m cannot provide the same degree of correlation with the surveyed map database. If examined individual parameter-wise, the ASTER 30 m dataset scores above or very close to the SRTM 90 m dataset in three out of the four parameters (i.e., for all three terrain parameters of mean elevation, relative relief and slope). The SRTM 90 m dataset is however, seemingly a better fit to derive streams from. The close correlation of the ASTER 30 m DEM dataset followed by that of the SRTM 90 m DEM dataset to the SoI topographical map database is also borne out by the correlation coefficients derived for each of the four parameters of mean elevation (Table 11), relative relief (Table 12), slope (Table 13) and drainage density (Table 14).

**Table 10:** Similarity in extracted values and isoline zones for the different parameters from various datasets in comparison to the T50A dataset

Sl. No.	Dataset	Difference class from T50A values	Average elevation			Relative relief			Slope			Drainage density			Averaged totals	Remarks
			Difference class range (m)	% Basin area	Total %	Difference class range (m)	% Basin area	Total %	Difference class range (degrees)	% Basin area	Total %	Difference class range (km / sqkm)	% Basin area	Total %		
1	A30	Immediate greater	−100 to 0	47.19	85.42	−250 to 0	40.06	99.26	−10 to 0	80.85	94.23	−2 to 0	11.55	50.20	82.28	Very high match
		Immediate lesser	0 to 100	38.23		0 to 250	59.20		0 to 10	13.38		0 to 2	38.65			
2	A90	Immediate greater	−500 to 0	25.60	52.87	−250 to 0	38.53	72.24	−10 to 0	38.65	58.52	−2 to 0	32.01	70.78	63.60	Low match
		Immediate lesser	0 to 500	27.27		0 to 250	33.71		0 to 10	19.87		0 to 2	38.77			
3	C30	Immediate greater	−100 to 0	50.75	65.55	−250 to 0	53.80	93.97	−10 to 0	70.93	82.41	−2 to 0	9.22	47.82	72.44	Moderate match
		Immediate lesser	0 to 100	14.80		0 to 250	40.17		0 to 10	11.48		0 to 2	38.60			
4	C90	Immediate greater	−500 to 0	27.04	54.62	−250 to 0	42.15	69.62	−10 to 0	37.41	50.80	−2 to 0	32.32	71.20	61.56	Low match
		Immediate lesser	0 to 500	27.58		0 to 250	27.47		0 to 10	13.39		0 to 2	38.88			
5	S90	Immediate greater	−100 to 0	48.74	86.23	−250 to 0	62.39	98.94	−10 to 0	80.68	87.65	−2 to 0	39.68	76.85	87.42	Very high match
		Immediate lesser	0 to 100	37.49		0 to 250	36.55		0 to 10	6.97		0 to 2	37.17			
6	T50D	Immediate greater	−100 to 0	60.61	94.12	−250 to 0	94.88	99.86	−10 to 0	82.81	84.76	−2 to 0	48.77	78.48	89.31	Highest match
		Immediate lesser	0 to 100	33.51		0 to 250	4.98		0 to 10	1.95		0 to 2	29.71			
7	T250A	Immediate greater	−500 to 0	46.95	74.96	−250 to 0	30.60	47.43	−10 to 0	31.43	52.04	−2 to 0	48.55	71.57	61.50	Lowest match
		Immediate lesser	0 to 500	28.01		0 to 250	16.83		0 to 10	20.61		0 to 2	23.02			
8	T250D	Immediate greater	−500 to 0	47.16	74.93	−250 to 0	38.64	58.79	−10 to 0	29.67	42.83	−2 to 0	41.85	79.06	63.90	Low match
		Immediate lesser	0 to 500	27.77		0 to 250	20.15		0 to 10	13.16		0 to 2	37.21			

Differences for each dataset are computed parameter-wise by subtracting from the corresponding values of that same parameter in the T50A database for each of the 1154 grids and isopleth classes are then demarcated on basis of this difference. Thus the immediate greater class (where T50A values are higher) and immediate lesser class (where T50A values are lower) together show areas which are more closely approximate to the values of the surveyed T50A database. If these two ranges combined, cover a greater proportion of the basin area, it implies then that that dataset matches well with the surveyed topographical map database, in terms of the parameters extracted and their ensuing isopleth zones. Perfect matching would give a narrow difference class range around zero covering the total basin area

**Table 11:** Correlation coefficients for grid-wise mean elevation values extracted from different datasets

	A30	A90	C30	C90	S90	T50D	T250D	T50A	T250A
A30	1.00								
A90	0.76	1.00							
C30	1.00	0.76	1.00						
C90	0.77	1.00	0.77	1.00					
S90	1.00	0.76	1.00	0.77	1.00				
T50D	1.00	0.76	0.99	0.77	1.00	1.00			
T250D	0.92	0.74	0.92	0.75	0.92	0.93	1.00		
T50A	0.99	0.76	0.99	0.77	0.99	0.99	0.92	1.00	
T250A	0.92	0.74	0.92	0.75	0.92	0.92	1.00	0.92	1.00

**Table 12:** Correlation coefficients for grid-wise relative relief values extracted from different datasets

	A30	A90	C30	C90	S90	T50D	T250D	T50A	T250A
A30	1.00								
A90	0.04	1.00							
C30	0.80	0.02	1.00						
C90	0.03	0.78	0.03	1.00					
S90	0.95	0.02	0.77	0.03	1.00				
T50D	0.79	0.03	0.66	0.02	0.80	1.00			
T250D	0.13	−0.07	0.08	−0.13	0.11	0.08	1.00		
T50A	0.76	0.04	0.62	0.02	0.76	0.93	0.09	1.00	
T250A	0.14	−0.08	0.09	−0.13	0.12	0.09	0.92	0.09	1.00

**Table 13:** Correlation coefficients for grid-wise slope values extracted from different datasets

	A30	A90	C30	C90	S90	T50D	T250D	T50A	T250A
A30	1.00								
A90	0.00	1.00							
C30	0.82	0.02	1.00						
C90	0.01	0.80	0.04	1.00					
S90	0.97	−0.01	0.82	0.01	1.00				
T50D	0.82	−0.01	0.71	0.01	0.84	1.00			
T250D	0.08	−0.13	0.05	−0.17	0.07	0.07	1.00		
T50A	0.65	0.00	0.53	0.01	0.66	0.79	0.03	1.00	
T250A	0.07	−0.12	0.03	−0.15	0.06	0.06	0.82	0.03	1.00

**Table 14:** Correlation coefficients for grid-wise drainage density values extracted from different datasets

	A30	A90	C30	C90	S90	T50D	T250D	T50A	T250A
A30	1.00								
A90	−0.03	1.00							
C30	0.83	0.00	1.00						
C90	−0.05	0.87	−0.02	1.00					
S90	0.85	−0.01	0.81	−0.03	1.00				
T50D	0.68	−0.02	0.68	−0.04	0.76	1.00			
T250D	0.28	−0.01	0.25	−0.05	0.27	0.38	1.00		
T50A	0.27	−0.03	0.23	−0.03	0.21	0.36	0.34	1.00	
T250A	0.23	−0.08	0.21	−0.09	0.20	0.31	0.62	0.42	1.00

## Conclusions

 Digital Elevation Models (DEMs) have been a subject of increasing attention and utilization in the last few decades because of the relative ease in delineation, extraction and calculation of various drainage and terrain morphometric parameters from them. Keeping this fact in mind, the present study was carried out in order to find the best possible DEM for computing the morphometric attributes of drainage basins from, especially in terrains that are difficult to survey or access. After analyzing the different morphometric parameters derived from these DEMs, it can be said that the DEMs derived from the 1:50,000 topographical map and ASTER GDEM datasets are relatively more accurate and consistent. They also exhibit a certain degree of proximity to the surveyed topographical map data. If 1:50,000 scale topographical maps of an area are not available, then the ASTER GDEM 30 m followed by the 4th generation SRTM DEM 90 m provides viable alternatives to analyse the terrain attributes of the area. While India’s indigenous and freely available Cartosat-1 DEM 30 m is unable to match the accuracy and consistency of the results produced by ASTER GDEM 30 m and SRTM DEM 90 m for this study area, the difference or deficiency is however lesser than those for resampled DEMs or DEMs prepared from smaller scale 1:250,000 scale topographical maps. The 30 m ASTER DEM also proves to be viable in examining terrains at even larger scales of 1:25,000; since topographical maps at this scale are rarely available for this country, due to an incomplete coverage. For large areas, where a greater numbers of maps are involved, these DEM datasets provide a relatively quicker pathway to topographic and drainage analysis.

DEM usage always comes with some caveats however. Sharma et al. (2009) while working on contour interpolated DEMs, postulated that grid size plays an important role in measuring the vertical accuracy of the DEMs. Furthermore, the generation of DEMs from topographical sheets can induce errors or omissions in scanning, georeferencing and digitisation, all of which may affect the resultant output DEM quality and the stream network information derived from it. This is corroborated by Ahmed et al. (2010) while working on the Bandihole Sub-watershed in Karnataka, India. However, in the present study, although the SRTM and ASTER datasets provide substantial results, the Cartosat-1 dataset does not provide similarly reliable information. Inherent limitations of the Cartosat-1 dataset might have played a part in reducing the accuracy of the first generation of this Cartosat-1 DEM.

## Abbreviations

A30: ASTER 30 m DEM dataset
A90: Resampled ASTER 90 m DEM dataset
ASTER: Advanced Spaceborne Thermal Emission and Reflection Radiometer
ASTER GDEM: Advanced Spaceborne Thermal Emission and Reflection Radiometer Global Digital Elevation Model
C30: CartoDEM 30 m DEM dataset
C90: Resampled CartoDEM 90 m DEM dataset
CartoDEM: Cartosat-1 Digital Elevation Model
DEM: Digital Elevation Model
FCC: False Colour Composite
GCP: Ground Control Point
GLOBE: Global Land 1 km-Base Elevation Project
GTOPO-30: Global Topography in 30 arc-sec
IRS: Indian Remote Sensing Satellite
ISRO: Indian Space Research Organisation
km: kilometre
LISS: Linear Imaging Self Scanning
m: metre
mm: millimetre
NW: North West
NRSC: National Remote Sensing Centre
R.F.: Representative Fraction
S90: SRTM 90 m DEM dataset
SCA: Specific Contributing Area
SE: South East
SoI: Survey of India
sq km: square kilometre
SRTM: Shuttle Radar Topography Mission
SRTM DEM: Shuttle Radar Topography Mission Digital Elevation Model
T250A: Actual Digitised Dataset from R.F. 1:250,000 USAMS Topographical Map
T250D: Prepared DEM Dataset from T250A Database
T50A: Actual Digitised Dataset from R.F. 1:50,000 SoI Topographical Map
T50D: Prepared DEM Dataset from T50A Database
TIN: Triangulated Irregular Network
USAMS: United States Army Map Service
USGS: United States Geological Survey
WGS84: World Geodetic System 1984
